# Relaxin/insulin-like family peptide receptor 4 (*Rxfp4*) expressing hypothalamic neurons modulate food intake and preference in mice

**DOI:** 10.1016/j.molmet.2022.101604

**Published:** 2022-09-30

**Authors:** Jo E. Lewis, Orla RM. Woodward, Danaé Nuzzaci, Christopher A. Smith, Alice E. Adriaenssens, Lawrence Billing, Cheryl Brighton, Benjamin U. Phillips, John A. Tadross, Sarah J. Kinston, Ernesto Ciabatti, Berthold Göttgens, Marco Tripodi, David Hornigold, David Baker, Fiona M. Gribble, Frank Reimann

**Affiliations:** 1 Wellcome Trust – MRC Institute of Metabolic Science Metabolic Research Laboratories, Addenbrooke's Hospital, Hills Road, Cambridge, CB2 0QQ, UK; 2 Department of Physiology, Development and Neuroscience, University of Cambridge, Cambridge, UK; 3 Department of Pathology, University of Cambridge, Cambridge CB2 1QP, UK; 4 Department of Haematology, Wellcome and MRC Cambridge Stem Cell Institute, University of Cambridge, Cambridge, UK; 5 MRC Laboratory of Molecular Biology, Neurobiology Division, Francis Crick Avenue, Cambridge CB2 0QH, UK; 6 Research and Early Development Cardiovascular, Renal and Metabolism (CVRM), BioPharmaceuticals R&D, AstraZeneca Ltd, Cambridge, UK

**Keywords:** RXFP4, Insl5, CNS, VMH, Food intake

## Abstract

**Objective:**

Insulin-like peptide 5 (INSL5) signalling, through its cognate receptor relaxin/insulin-like family peptide receptor 4 (RXFP4), has been reported to be orexigenic, and the high fat diet (HFD) preference observed in wildtype mice is altered in *Rxfp4* knock-out mice. In this study, we used a new *Rxfp4*-Cre mouse model to investigate the mechanisms underlying these observations.

**Methods:**

We generated transgenic *Rxfp4*-Cre mice and investigated central expression of *Rxfp4* by RT-qPCR, RNAscope and intraparenchymal infusion of INSL5. *Rxfp4*-expressing cells were chemogenetically manipulated in global Cre-reporter mice using designer receptors exclusively activated by designer drugs (DREADDs) or after stereotactic injection of a Cre-dependent AAV-DIO-Dq-DREADD targeting a population located in the ventromedial hypothalamus (RXFP4^VMH^). Food intake and feeding motivation were assessed in the presence and absence of a DREADD agonist. *Rxfp4*-expressing cells in the hypothalamus were characterised by single-cell RNA-sequencing (scRNAseq) and the connectivity of RXFP4^VMH^ cells was investigated using viral tracing.

**Results:**

*Rxfp4-*Cre mice displayed Cre-reporter expression in the hypothalamus. Active expression of *Rxfp4* in the adult mouse brain was confirmed by RT-qPCR and RNAscope. Functional receptor expression was supported by cyclic AMP-responses to INSL5 application in *ex vivo* brain slices and increased HFD and highly palatable liquid meal (HPM), but not chow, intake after intra-VMH INSL5 infusion. scRNAseq of hypothalamic RXFP4 neurons defined a cluster expressing VMH markers, alongside known appetite-modulating neuropeptide receptors (*Mc4r, Cckar* and *Nmur2*). Viral tracing demonstrated RXFP4^VMH^ neural projections to nuclei implicated in hedonic feeding behaviour. Whole body chemogenetic inhibition (Di-DREADD) of *Rxfp4*-expressing cells, mimicking physiological INSL5-RXFP4 Gi-signalling, increased intake of the HFD and HPM, but not chow, whilst activation (Dq-DREADD), either at whole body level or specifically within the VMH, reduced HFD and HPM intake and motivation to work for the HPM.

**Conclusion:**

These findings identify RXFP4^VMH^ neurons as regulators of food intake and preference, and hypothalamic RXFP4 signalling as a target for feeding behaviour manipulation.

## Highlights

• The cognate receptor for INSL5, RXFP4, is expressed in the CNS of mice.
• Functional receptor expression was supported by cAMP-responses in acute brain slices.
• Intra-VMH infusion of INSL5 increased HFD and HPM intake in mice.
• Viral tracing and RNASeq suggest a role in hedonic feeding behaviour.
• Chemogenetic strategies at whole-body, or specifically within VMH, confirm phenotype.

## 1 Introduction

Relaxin/insulin-like family peptide receptor 4 (RXFP4) is the cognate receptor for insulin-like peptide 5 (INSL5), a member of the relaxin/insulin-like peptide family [[1], [2], [3]]. We previously reported an orexigenic effect of exogenously applied INSL5 in mice [4]. More recently, we observed a transient increase in food intake when *Insl5*-expressing cells were chemogenetically activated [5]. This was only revealed when the dominant anorexigenic action of peptide YY (PYY), co-secreted from the same cell population in the distal large intestine [6], was blocked with a NPY2R antagonist [5]. Reports that *Insl5* knockout (*Insl5*^−/−^) mice do not display an observable feeding phenotype and that, in some studies, pharmacological administration of INSL5 (both native and PEGylated forms) failed to increase food intake in lean and obese mice [7,8], have shed doubt on whether INSL5 plays a physiologically relevant role in the control of food intake. However, *Rxfp4* knockout (*Rxfp4*^−/−^) mice exhibit shorter meal durations, particularly when fed a high fat diet (HFD), and lack the normal preference for HFD over standard chow diet observed in wildtype mice [4]. We therefore consider RXFP4 to be a potential target receptor for the manipulation of feeding behaviour.

RXFP4 is a G_αi/o_-coupled receptor identified in 2003 through its homology to relaxin/insulin-like peptide receptor 3 (RXFP3) [9,10]. Binding of INSL5 activates downstream signalling pathways including phosphorylation of ERK1/2, Akt, p38MAPK and S6RP and reduces cytosolic cAMP levels through inhibition of adenylyl cyclase [2]. In addition to the selective ligand INSL5, relaxin-3 can also activate RXFP4, stimulating comparable signalling pathways [2,11]. *Rxfp4* mRNA expression has been reported in the colon, kidney, heart, liver, testes and ovary of both mice and humans [1,10,11]. To identify and manipulate *Rxfp4*-expressing cells, we developed a new transgenic mouse model in which Cre-recombinase expression is driven by the *Rxfp4* promoter (*Rxfp4*-Cre). Using this model, we found clear Cre-dependent reporter expression within the central nervous system (CNS), including the ventromedial hypothalamus (VMH), a finding we confirmed via RT-qPCR and RNAScope. This contrasts with our previous study, in which we failed to detect *Rxfp4* expression in the central nervous system by RT-qPCR, and in which intracerebroventricular administration of INSL5 failed to increase food intake significantly [4]. Revisiting the potential central role of RXFP4 in food intake regulation, we observed that infusion of INSL5 directly into the VMH induced a significant orexigenic response when mice were offered a HFD or a highly palatable liquid Ensure test meal (HPM), but not when offered a standard chow diet. We therefore used the *Rxfp4*-Cre mouse model to explore the role of *Rxfp4*-expressing cells in modulating food intake and preference.

## 2 Results

**Figure 1:**
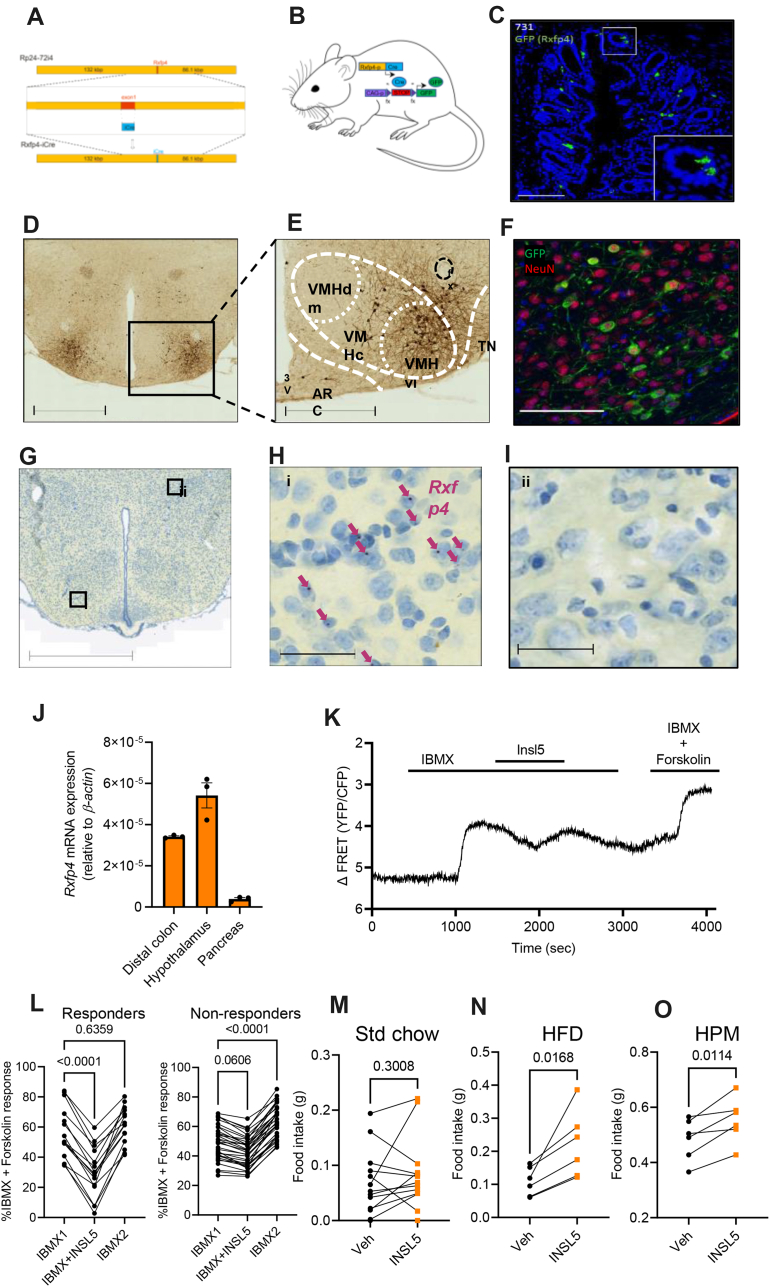
***Rxfp4*is expressed in the central nervous system.** (A) Scaled schematic of the bacterial artificial chromosome used to make *Rxfp4*-Cre mice. (B) Crossing of *Rxfp4*-Cre mice with GFP-based reporter mice (EYFP or GCaMP3) used to detect *Rxfp4* expression in this figure through GFP immunohistochemistry. (C) Representative section from the colon of RXFP4^EYFP^ mice demonstrating *Rxfp4* expression in epithelial cells. Scale bar = 100 μm. (D) and (E) Coronal section of RXFP4^GCaMP3^ mice showing distinct *Rxfp4*-expressing cell clusters in the ventrolateral part of the ventromedial hypothalamus (VMHvl) and adjacent central VMH (VMHc) and tuberal nucleus (TN). Scale bars = 1 mm and 500 μm respectively. (F) Co-staining for DAPI (blue), GFP (green) and NeuN (red) in the VMH of RXFP4^GCaMP3^ mice. Scale bar = 100 μm. (G–I) Coronal sections of C57Bl6 mice labelled for *Rxfp4* mRNA in the VMH using RNAscope (G) and (H), including negative area for comparison (I). Scale bar = 1 mm (G) and 50 μm in the enlarged images (H and I). (J) RT-qPCR to determine *Rxfp4* mRNA in the distal colon, hypothalamus and pancreas of mice. (K) Representative trace of a recorded cell in a RXFP4^CAMPER^ slice responding to IBMX (100 μM) in the absence and presence of INSL5 (100 nM) and Forskolin (10 μM), as indicated. 15 out of 48 cells responded to INSL5 with the same pattern (n = 5 mice). (L) Responses to IBMX (100 μM) in the absence and presence of INSL5 (100 nM) expressed in percentage of IBMX (100 μM) and Forskolin (10 μM) maximum response in responding and non-responding cells (n = 5 mice). M, N, O) Food intake of wildtype mice following intra-VMH infusion of vehicle/INSL5 (5 μg) of (M) standard chow (t = 1.081, p = 0.3008 n = 13), (N) HFD (45%) (t = 3.529 n = 6, p = 0.0168) and (O) liquid Ensure (HPM) (t = 3.902, p = 0.0114 n = 6, paired two-tailed t test). Animals adapted to the appearance of test meal over the course of two weeks prior to surgery and recovery, testing conducted between 12:00 and 15:00.

To investigate a possible role of *Rxfp4*-expressing cells in feeding control, we generated a new bacterial artificial chromosome (BAC) transgenic mouse (*Rxfp4*-Cre) model in which Cre-recombinase is expressed under the control of the *Rxfp4*-promoter (Figure 1A). By crossing *Rxfp4*-Cre mice with fluorescent protein reporter mice (e.g. Rosa26 fxSTOPfx-EYFP (RXFP4^EYFP^)) (Figure 1B) we observed *Rxfp4* dependent expression in the colon (Figure 1C), consistent with previous reports, but also detected reporter expression in the CNS (Suppl. Fig. 1) by GFP immunohistochemistry. Within the CNS we observed reporter expression in cells of the VMH, the accessory olfactory bulb, septofimbrial nucleus, retrosplenial cortex, and the mammillary body medial and lateral parts, as well as in a lower density of cells in the substantia innominata, cortical and central amygdala, periaqueductal grey, the lateral dorsal tegmental nucleus, nucleus of the tractus solitarius, area postrema and spinal trigeminal tract of the hindbrain (Suppl. Fig. 1). GFP-positive nerve fibres were also detected in the bed nucleus of the stria terminalis, anterodorsal thalamic nucleus, mammillothalamic tract, suprachiasmatic nucleus, paraventricular hypothalamus and ventral tegmental nucleus of Gudden (Suppl. Fig. 1). Closer examination of the VMH using RXFP4^GCaMP3^ mice revealed transgene expression in the central and ventrolateral parts (VMHc and VMHvl, respectively) extending into the adjacent tuberal nucleus (TN) (Figure 1D and E). The VMH is a central hub for the regulation of energy balance and integration of diverse nutritionally regulated hormonal and synaptic inputs, and the adjacent TN has also been implicated in feeding behaviour [12,13]. In the VMH, co-staining with the neuronal marker NeuN demonstrated GFP expression in mature *Rxfp4* positive neurons (Figure 1F). Active transcription of *Rxfp4* mRNA in the adult mouse VMH was confirmed by RNAscope, at the low abundance expected for G protein-coupled receptors (Figure 1G–I), and RT-qPCR, with levels comparable to the distal colon, a known site of *Rxfp4* expression (Figure 1J). To test the functional activity of the RXFP4-INSL5 pathway in the *Rxfp4*-expressing cells in the VMH (RXFP4^VMH^), we performed real time monitoring of cAMP levels in RXFP4^VMH^ cells using *ex vivo* CAMPER [14] imaging in hypothalamic slices from *Rxfp4-*Cre x CAMPER (RXFP4^CAMPER^) mice. We found that 15 out of 48 YFP-positive cells showed a clear reduction in the ΔFRET ratio (YFP/CFP), reflecting a reduction in cAMP levels, in response to INSL5 (Figure 1K and L), whilst the remaining cells displayed no obvious change in FRET signal in response to INSL5 addition, within the limits of the assay sensitivity. To test the functional significance of the RXFP4^VMH^ cells *in vivo*, we treated wildtype ad libitum (AL) fed lean mice with an intra-VMH infusion of INSL5 after a 2 h fast. During the light phase, INSL5 had no effect on standard (std) chow intake (Figure 1M) but in mice habituated to the appearance of a HFD or HPM for 1 h, treatment with INSL5 significantly increased HFD and HPM intake compared to the vehicle control treatment (Figure 1N and O).

**Figure 2:**
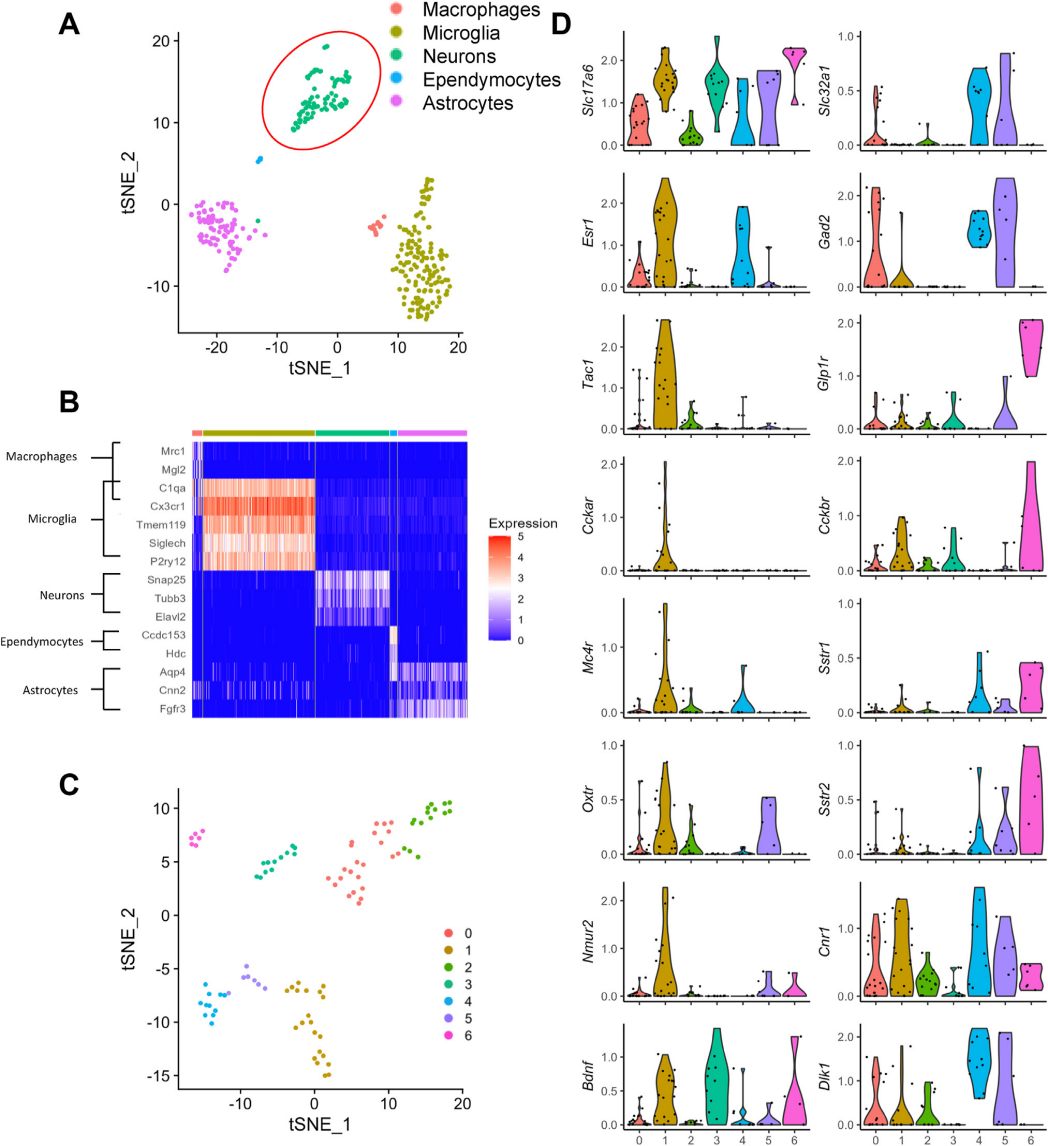
**Transcriptomic profiling of hypothalamic *Rxfp4*-expressing cells by scRNAseq**. (A) and (B) tSNE visualisation of 350 hypothalamic *Rxfp4*-expressing cells revealed five clusters (A). Cell types were assigned according to the expression of a combination of canonical cell-type markers genes (B). The red circle indicates the neuronal cluster used in further analysis. (C) tSNE visualisation of the 95 neuronal cells revealed 7 sub-clusters. (D) Violin plots showing expression of marker genes associated with multiple neuronal cell types. All gene expression counts are log-normalised with scale-factor = 10^4^.

As intra-VMH INSL5 significantly modulated feeding behaviour, we further characterised the *Rxfp4*-expressing cells in this region. Initially, we generated a single cell resolution transcriptomic profile of *Rxfp4*-expressing cells in the hypothalamus. Fluorescent cell populations from the hypothalami of twelve RXFP4^EYFP^ mice were purified by fluorescence-activated cell sorting (FACS) and their transcriptomes analysed by single-cell RNA-sequencing (scRNAseq). After data normalisation, 350 cells passed quality control filtering. Graph-based clustering analysis revealed that the filtered cells separated into five populations (Figure 2A). Cluster identities were assigned based on the expression patterns of canonical cell-type markers, identifying microglia (*Tmem119*, *Siglech*, *P2ry12*), neuronal cells (*Snap25, Tubb3, Elavl2*) and astrocytes (*Aqp4, Cnn2, Fgfr3*), together with smaller clusters of macrophages (*Mrc1*, *Mgl2*) and ependymocytes (*Ccdc153*, *Hdc*) (Figure 2B). As IHC had suggested neuronal *Rxfp4* expression (Figure 1F) and hypothalamic neurons are known to modulate feeding behaviour, we analysed the neuronal cluster in more detail, identifying seven subclusters (Figure 2C). *Rxfp4*-positive neurons expressed markers for both GABAergic (*Slc32a1*) and glutamatergic (*Slc17a6*) cells (Figure 2D). Cluster 1 was enriched in markers previously associated with an estrogen receptor (*Esr1*)-positive VMHvl neuronal population [[15], [16], [17]], including preprotachykinin-1 (*Tac-1*), oxytocin receptor (*Oxtr*), cholecystokinin receptor A (*Cckar*), melanocortin 4 receptor (*Mc4r*) and neuromedin U receptor 2 (*Nmur2*) (Figure 2D), suggesting crosstalk with known food regulatory networks. Receptors for other established feeding-neuromodulators, like glucagon-like peptide-1 receptor (*Glp1r*) and cholecystokinin receptor B (*Cckbr*), were preferentially expressed in cluster 6 (Figure 2D).

**Figure 3:**
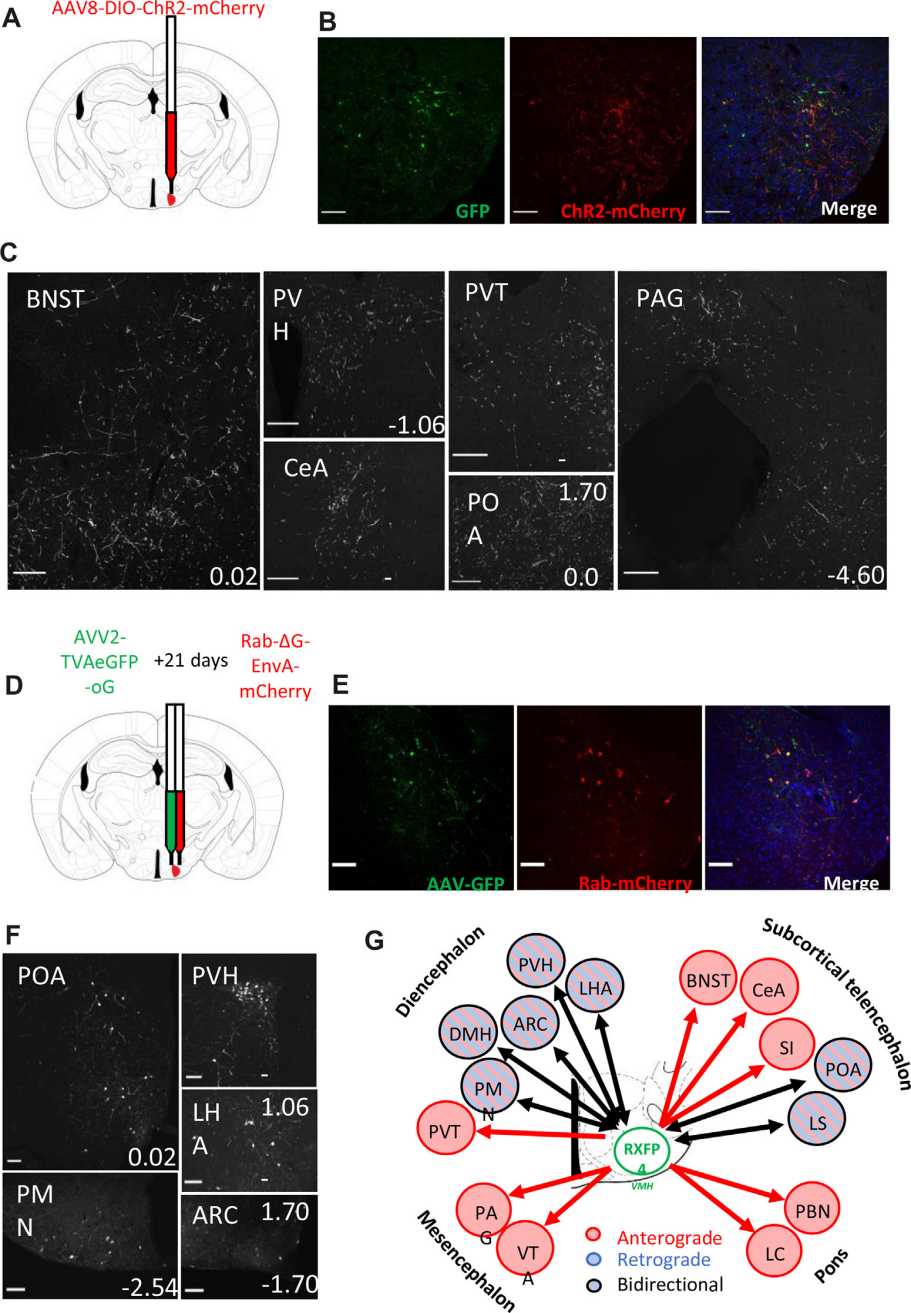
**Circuit mapping of hypothalamic *Rxfp4*-expressing cells.** (A) Schematic illustrating unilateral microinjection of Cre-dependent rAAV8-DIO-hChR2(H134R)-mCherry into the VMH of RXFP4^GCaMP3^ mice. (B) Immunofluorescence images demonstrating co-localisation of GFP (green) and ChR2-mCherry (red) starter cells in the target region at A/P −1.7 mm from bregma. (C) Representative images showing ChR2-mcherry-immunoreactive axon terminals in various brain regions (n = 3). For each image, distance from bregma (in mm) is indicated at the bottom right. Scale bars = 100 μm. 40× magnification. (D) Schematic illustrating unilateral microinjection of Cre-dependent AVV2-TVAeGFP-oG into the VMH of *Rxfp4*-Cre mice followed by a unilateral microinjection of Rab-ΔG-EnvA-mCherry 21 days later. (E) Immunofluorescence images demonstrating the colocalisation of GFP (green) and Rab-mCherry (red) starter cells in the target region A/P −1.7 mm from bregma. (F) Representative immunofluorescence images showing Rab-mCherry-immunoreactive cell bodies in various brain regions (n = 3). For each image distance from bregma (in mm) is indicated at the bottom right. 20× magnification. (G) Schematic illustrating the regions positive for anterograde projections (red arrows), retrograde projections (blue arrows) or bilateral projections (black arrows) from RXFP4^VMH^ cells. Abbreviations: ARC: arcuate nucleus; BNST: bed nucleus of the stria terminalis; CeA: central amygdala; LHA: lateral hypothalamic area; LS: lateral septum; PAG: periaqueductal grey; PMN: premammillary nucleus; POA: Preoptic area; PVH: paraventricular hypothalamus; PVT: paraventricular thalamic nucleus; VMH: ventromedial hypothalamus.

We subsequently aimed to establish the neuronal circuitry around RXFP4^VMH^ cells. Anterograde projections were mapped by stereotactically injecting Cre-dependent rAAV8-ChR2-mCherry into the VMH of RXFP4^GCaMP3^ mice [18] (Figure 3A and B). Axonal transport of the ChR2-mCherry fusion protein revealed RXFP4^VMH^ projections to multiple regions including the bed nucleus of the stria terminalis (BNST), preoptic area (POA), anteroventral periventricular nucleus (AVPV), arcuate nucleus (ARC), paraventricular hypothalamus (PVH), central nucleus of the amygdala (CeA), periaqueductal grey (PAG, dorsomedial and lateral) and parabrachial nucleus (PBN, lateral and medial) (Figure 3C, Suppl. Fig. 3). Retrograde projections were assessed after AAV2-TVAeGFP-oG injection into the VMH of *Rxfp4*-Cre mice followed by Rab-ΔG-EnvA-mCherry [19] injection 21 days later (Figure 3D and E). The retrograde monosynaptic transport of Rab-mCherry labelled inputs from several nuclei established in feeding regulation, including the ARC, DMH and PVH (Figure 3F). The neuronal circuitry surrounding RXFP4^VMH^ cells is summarised in Figure 3G.

**Figure 4:**
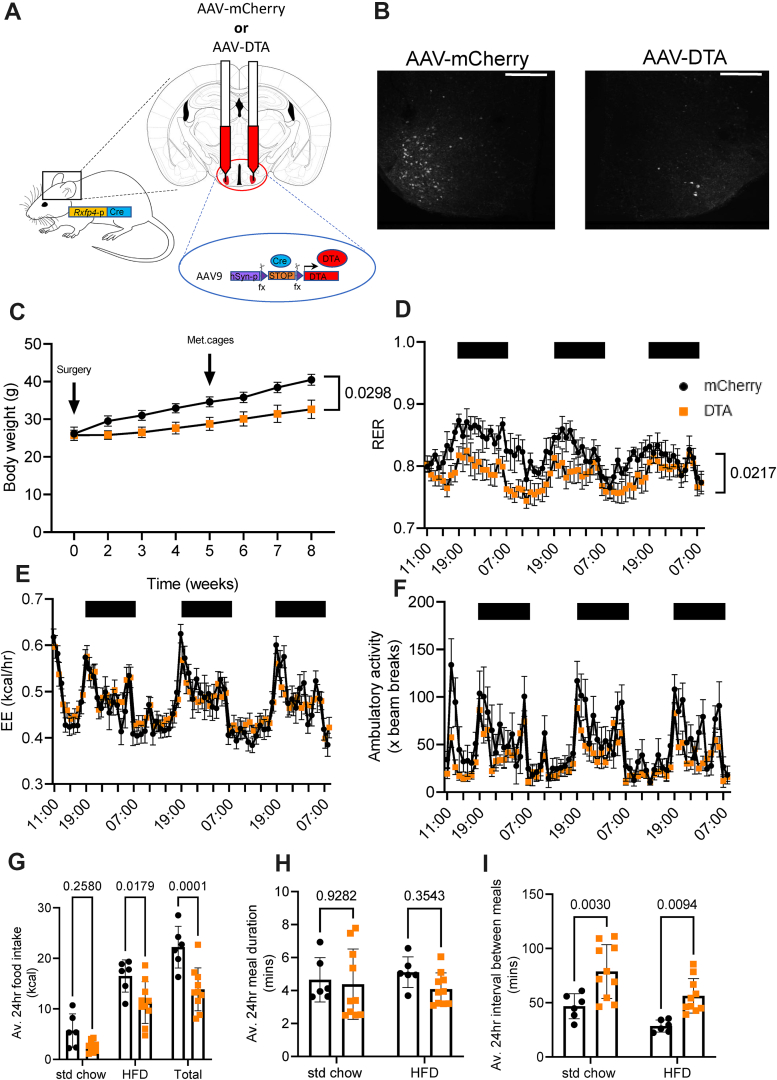
**VMH ablation of *Rxfp4*-expressing cells regulates food intake and body weight**. (A) Cre-dependent rAAV-mCherry (control) or Cre-dependent rAAV-DTA were bilaterally injected into the VMH of RXFP4^GCaMP3^ mice. (B) Targeting efficiency was confirmed post perfusion fixation by GFP-immunohistochemistry. Representative images showing ablation in the VMH. (C) Body weight following exposure to standard chow and HFD in parallel (effect of time [F (1.423, 22.76) = 44.45, p < 0.0001], effect of treatment [F (1,16) = 5.687, p = 0.03) (n = 8–10 per group). (D) RER effect of time [F (6.263, 87.69) = 3.368, p = 0.0044)], effect of treatment [F (1, 14) = 6.669, p = 0.02]. (E) Energy expenditure effect of time [F (9.339,130.7) = 20.34, p < 0.0001], effect of treatment [F (1,14) = 0.004, p = 0.95] and (F) ambulatory activity effect of time [F (7.723, 100.4) = 11.69, p < 0.0001], effect of treatment [F (1,16) = 3.270, p = 0.09] of mice housed in metabolic cages 5 weeks post-surgery. (G) Average 24 h food intake (effect of diet [F (2,42) = 61.97, p < 0.0001) and effect of treatment [F (1.42) = 28.27, p < 0.0001) (H) Average 24 h meal duration (effect of diet [F (1,28) = 0.02361, p = 0.88) and effect of treatment [F (1,28) = 1.393, p = 0.25) and (I) Average 24 h interval between meals (effect of diet [F (1,28) = 10.08, p = 0.0046) and effect of treatment [F (1,28) = 21.71, p < 0.0001) of mice treated with rAAV-mCherry or rAAV-DTA of RXFP4^VMHKO^ mice in metabolic chambers, when mice had access to a choice between std chow and a HFD (rAAV-mCherry n = 6, rAAV-DTA n = 10 respectively, group mean ± SEM, black circles rAAV-mCherry, orange squares rAAV-DTA). Scale bars = 400 μm.

Due to the previously reported altered feeding patterns and macronutrient preferences of *Rxfp4*^−/−^ mice [4], we further explored the physiology of the RXFP4^VMH^ population by ablating *Rxfp4*-expressing cells using rAAV-DTA injected into the VMH of RXFP4^GCaMP3^ mice (Figure 4A). Post-mortem histological analysis revealed a >90% reduction in RXFP4^VMH^ cells (identified by GFP immunohistochemistry) across the VMH (Figure 4B). In the 8 weeks following the surgery, the RXFP4^VMHKO^ mice gained less body weight compared to the control rAAV-mCherry injected mice (Figure 4C), when fed with a choice of standard chow and HFD in parallel. Five weeks post-surgery the mice were studied in metabolic cages. RXFP4^VMHKO^ mice had a lower respiratory exchange ratio (RER, Figure 4D), whilst energy expenditure (in kcal/h [Figure 4E] and using body weight as a covariate [Suppl. Fig. 4d]) and ambulatory activity (Figure 4F) were unaffected by ablation. Average 24 h food intake was significantly reduced, measured by ANCOVA using body weight as a covariate (see Suppl. Fig. 4f), a consequence of reduced HFD intake (Figure 4G). Meal duration was unaffected by treatment, however the interval between meals (Figure 4I) was significantly increased. These data demonstrate the importance of this neuronal population in governing long-term feeding behaviour. There was no observable difference between male and female animals (see suppl. Fig. 4a–i).

**Figure 5:**
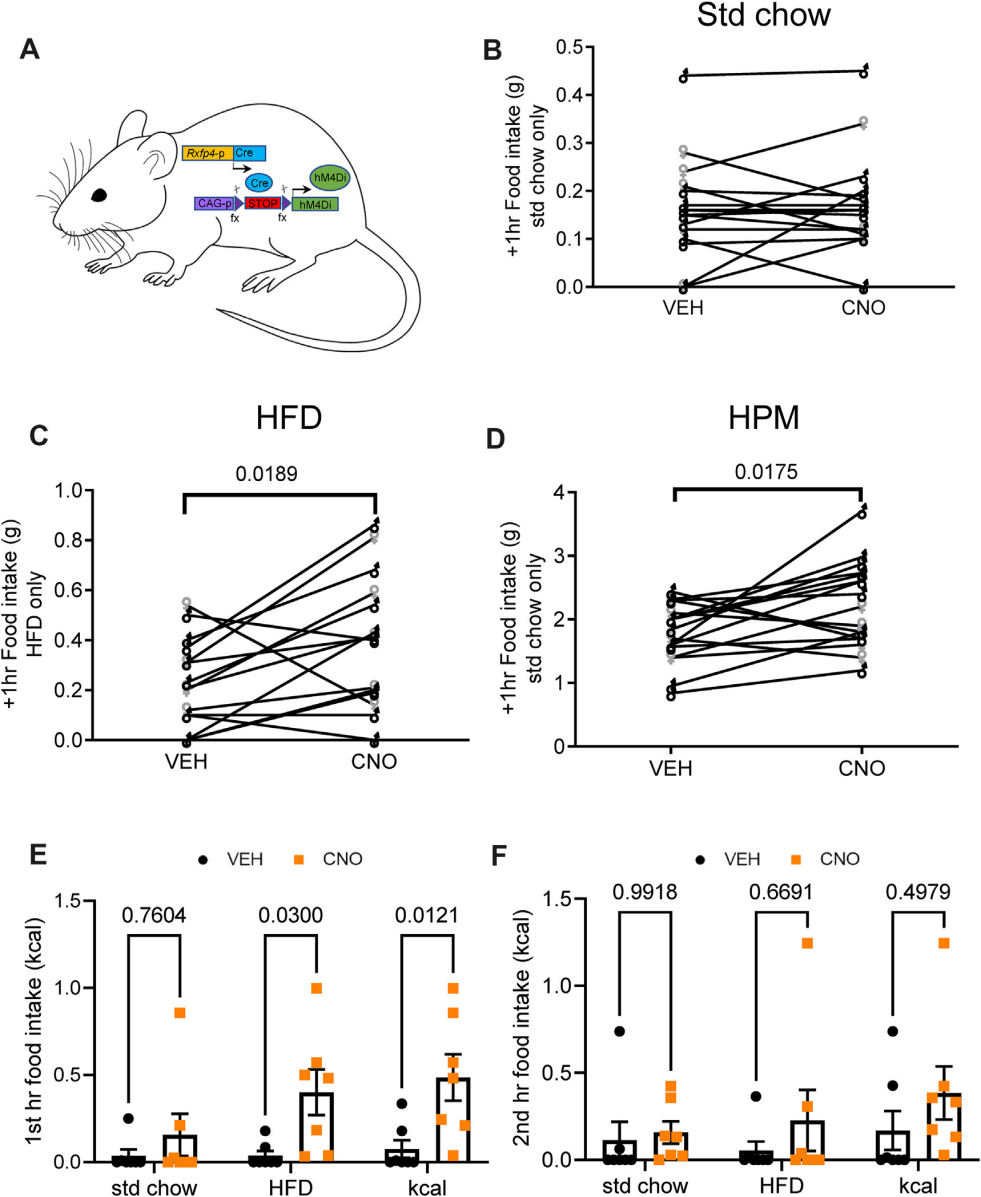
**CNO alters feeding and food preference in RXFP4^wb−Di^ mice**. (A) Schematic of RXFP4^wb−Di^ mouse model. B–D) Food intake in RXFP4^wb−Di^ mice (male ♂ (black), female ♀ (grey)) of (B) standard chow (t = 0.5536, p = 0.5875) (C) or HFD (45%) (t = 2.612, p = 0.019) (D) or liquid Ensure (HPM) (t = 2.648, p = 0018) 1 h post CNO/vehicle treatment at 11:00 (n = 17, paired two-tailed t test, animals adapted to the appearance of test meal over the course of two weeks, male ♂ (black), female ♀ (grey)). E) 1st hr (effect of treatment [F (1,36) = 14.86, p = 0.0005], std chow p = 0.7604, HFD p = 0.03, kcal p = 0.012) and (F) 2nd hour food (effect of treatment [F (1,36) = 2.219, p = 0.15], std chow p = 0.9918, HFD p = 0.6691, kcal p = 0.50) consumption after CNO (orange) or saline (black) injection at 11:00 in mice housed in metabolic chambers (n = 7, two-way ANOVA with Sidak's multiple-comparison test) when mice had a choice between standard chow or HFD.

Given that RXFP4^VHMKO^ mice ate less HFD, yet central Insl5 infusion, which we would predict to inhibit *Rxfp4*-expressing neurons, increased HFD intake, we investigated the effects of acute activation or inhibition of *Rxfp4*-expressing cells. Initially we used a whole-body hM4Di DREADD Cre-reporter (RXFP4^wb−Di^) to mimic the established RXFP4-signalling via pertussis-toxin sensitive Gi pathways [2] (Figure 5A). During the light phase, activation of Di in *Rxfp4*-expressing cells using the DREADD agonist clozapine-N-oxide (CNO) had no measurable effect on food intake in AL fed male and female mice (Figure 5B, as indicated). However, when animals were habituated to the appearance of a HFD or HPM for 1 h during the light phase, CNO application resulted in increased food intake (Figure 5C and D). These results were consistent with the response to infusion of INSL5 into the VMH. To investigate this further, we gave male mice housed in metabolic cages the choice between standard chow and HFD. CNO injection during the light phase significantly increased HFD but not chow intake in RXFP4^wb−Di^ mice (Figure 5E). This effect was transient (Figure 5F), consistent with the pharmacokinetics of CNO [20], and occurred without any significant differences in ambulatory activity or energy expenditure compared to the vehicle cross-over control (Suppl. Fig. 5a and b).

**Figure 6:**
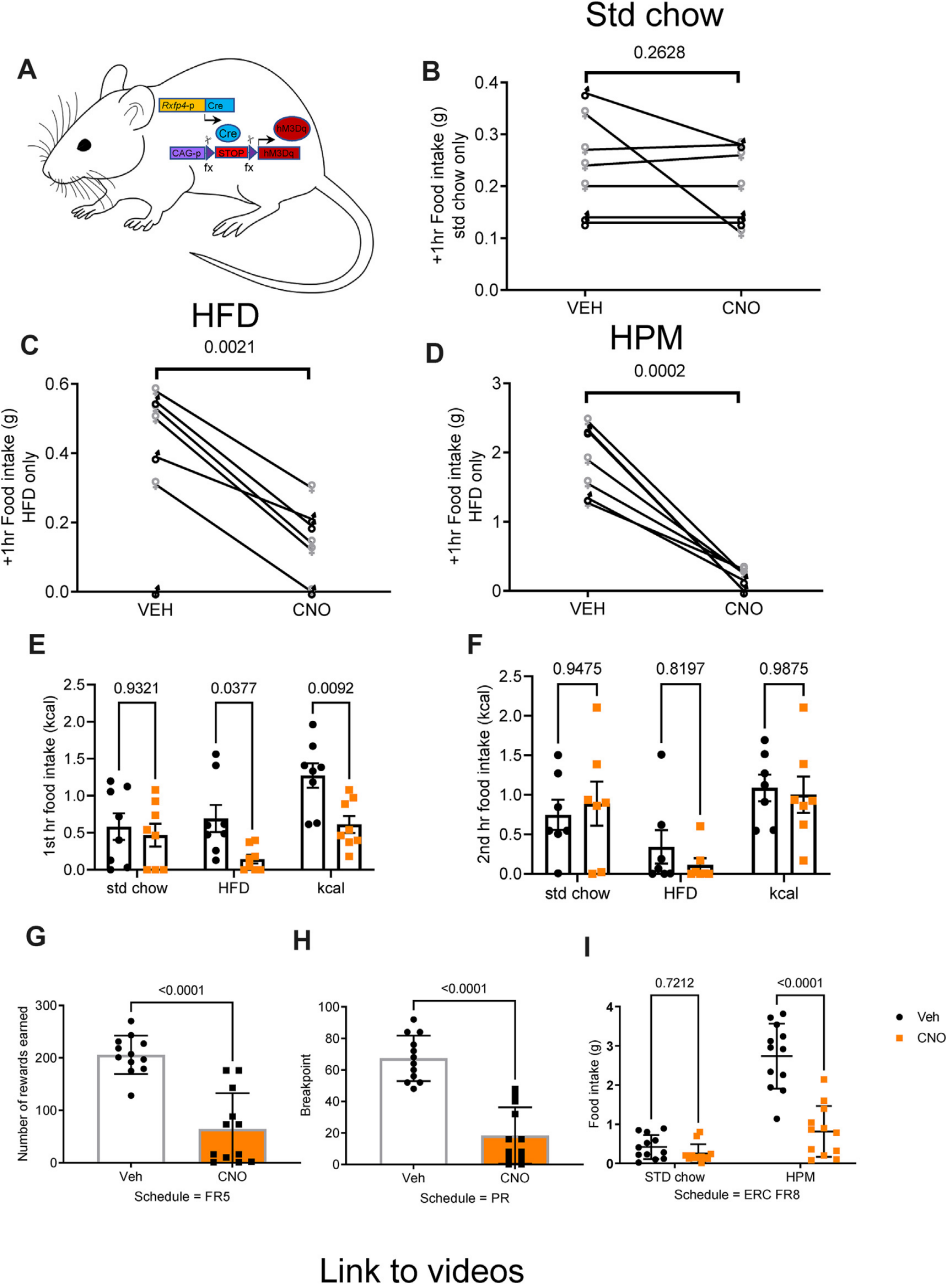
CNO alters feeding and food preference in RXFP4^wb−Dq^ mice. (A) Schematic of RXFP4^wb−Dq^ mouse model. B-D) Food intake of (B) standard chow (t = 0.1.235, p = 0.2628) (C) HFD (45%) (t = 5.136, p = 0.0021) and (D) liquid Ensure (HPM) (t = 7.725, p = 0.0002) 1 h post CNO/saline treatment at 19:00 (n = 7, paired two-tailed t test, animals adapted to the appearance of test meal over the course of two weeks in male and female mice, male ♂ (black), female ♀ (grey)). (E) 1st hour (effect of treatment [F (1,42) = 13.17, p = 0.0008, standard chow p = 0.9321, HFD p = 0.0377, kcal p = 0.009) and (F) 2nd hour (effect of treatment [F (1,36) = 0.1177, p = 0.7335], std chow p = 0.9475, HFD p = 0.8197, kcal p = 0.9875) food consumption after CNO (orange) or saline (black) injection at 19:00 in mice housed in metabolic chambers (n = 7, two-way ANOVA with Sidak's multiple-comparison test) when mice had access to a choice between standard chow and a HFD. (G–I) Performance parameters in mice trained in operant chambers when treated with CNO (orange) or vehicle (black): (G) Number of rewards earned in FR5 (n = 12, t = 6.874, p < 0.001, paired two-tailed t test). (H) Breakpoint in PR4 (number of target responses emitted by an animal in the last successfully completed trial, before session termination or 60 min time-out) (n = 12, t = 9.357, ∗∗∗p < 0.001, paired two tailed t test). (I) ERC performance post vehicle (black) or CNO (orange) treatment between 11 and 13:00 (n = 12, effect of treatment [F (1,44) = 41.38, p < 0.0001, std chow p = 0.72, HPM p < 0.0001, two-way ANOVA with Sidak's multiple-comparison test, animals calorically restricted to 95% BW).

We next investigated the effects of whole-body hM3Dq Cre-reporter activation in *Rxfp4*-expressing cells (RXFP4^wb−Dq^) (Figure 6A). After a 2 h brief fast, activation of Dq in *Rxfp4*-expressing cells at the onset of the dark phase had no measurable effect on the food intake of male and female mice offered only standard chow (Figure 6B, as indicated). However, when RXFP4^wb−Dq^ animals were habituated to the appearance of HFD or a HPM for 1 h at the onset of the dark phase, activation of Dq-expressing cells with CNO resulted in a marked reduction in HFD and HPM intake (Figure 6C and D). These results were consolidated in AL fed male animals tested in metabolic cages with parallel access to standard chow and HFD. RXFP4^wb−Dq^ activation in AL fed animals at the onset of the dark phase had no effect on standard chow intake, but significantly and transiently reduced HFD consumption (Figure 6E and F). RXFP4^wb−Dq^ activation also attenuated the increase in energy expenditure associated with the onset of the dark phase, however, there was no effect on ambulatory activity (Suppl. Fig. 5c and d).

To probe whether *Rxfp4*-expressing cells play a role in the motivational aspects of feeding, we calorically restricted male RXFP4^wb−Dq^ animals to 95% body weight and placed them in operant chambers. Mice were tested with a fixed ratio (FR) schedule, requiring 5 nose pokes to release a food reward (liquid Ensure, HPM), or a progressive ratio (PR) schedule requiring increasing number of nose pokes for each subsequently earned reward (in this case, +4, i.e. 1, 5, 9, 13, etc). RXFP4^wb−Dq^ mice treated with CNO completed fewer attempts under FR to earn individual Ensure rewards (Figure 6G). Under a PR schedule, they exhibited a reduced breakpoint, i.e. CNO-treated mice stopped working for the HPM-reward at lower ratios than when receiving vehicle treatment (Figure 6H). In an effort related choice (ERC) paradigm, where animals had the choice of working for a HPM (FR8, liquid Ensure) or consuming freely available standard chow, CNO treatment reduced HPM consumption (Figure 6I). However, animals consumed similar amounts of standard chow and displayed otherwise normal behaviour (supplementary video 1), suggesting that activation of *Rxfp4*-expressing cells reduced motivation for the HPM rather than inducing generalised malaise.

The following is/are the supplementary data related to this article:

Video 1 2 Video 1

Video 2 3 Video 2

**Figure 7:**
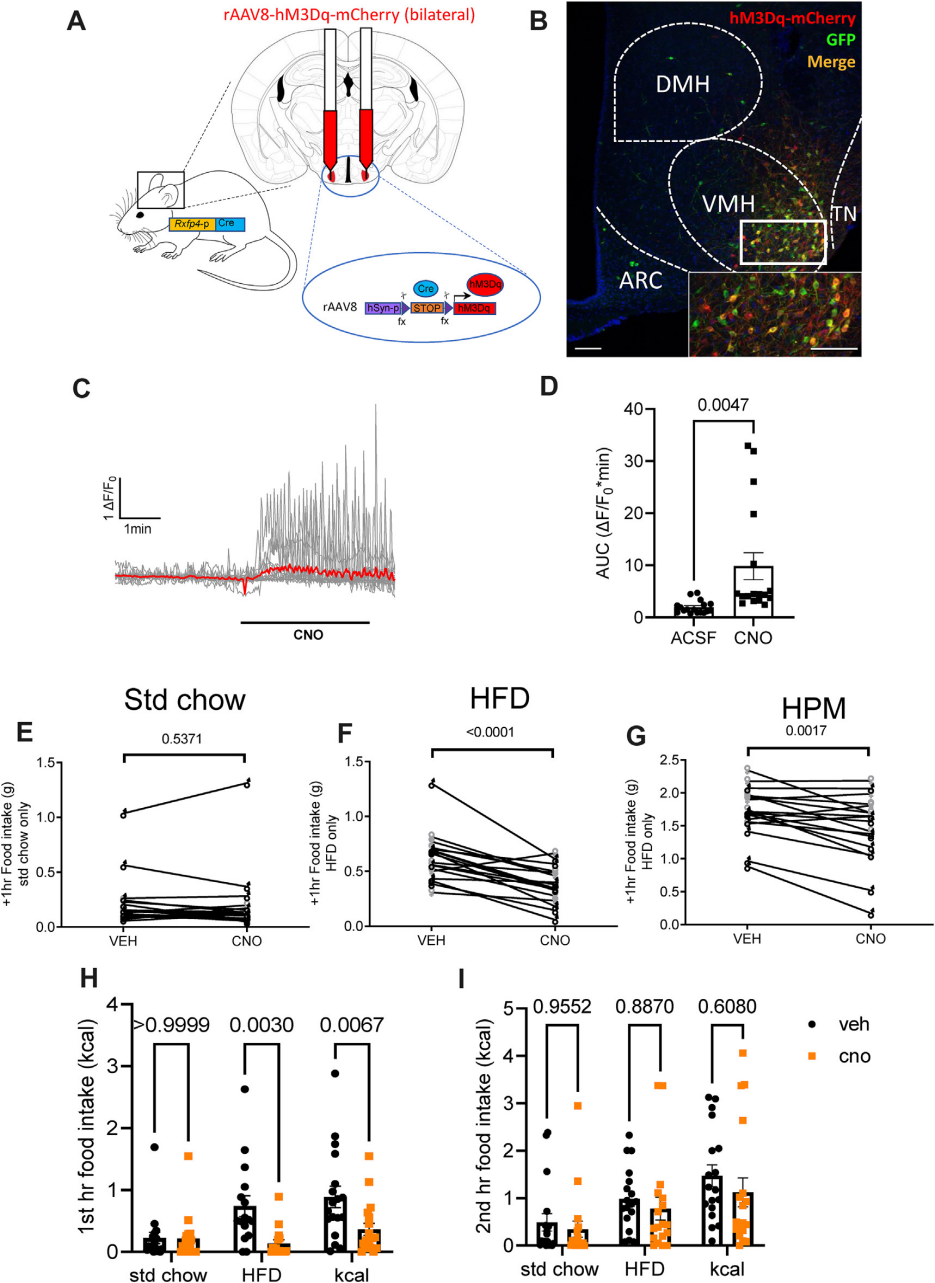
**VMH*Rxfp4*-expressing neurons regulate food preference**. (A) Cre-dependent rAAV-hM3Dq-mCherry was bilaterally injected into the VMH of *Rxfp4*-Cre mice. (B) Targeting efficiency was confirmed post perfusion fixation. Representative image showing ARC, DMH, VMH and TN with only VMH demonstrating rAAV-hM3Dq-mCherry expression. (C) Representative images of RXFP4^VMHDq^ cells before, during and after bath applied CNO (15 μM) showing somatic GCaMP3 fluorescence (top panel), with traces from individual cells (bottom panel, mean intensity shown in red). (D) Quantification of responses showing median AUC before and during bath applied CNO (t = 3.285, p = 0.0047 paired two-tailed t test, n = 17 cells from 3 mice). (E) and (F) Food intake of (E) standard chow (t = 0.63, p = 0.5371) (F) HFD (45%) (t = 5.630, p < 0.0001) and (G) liquid Ensure (HPM) (t = 3.714, 0.0017) 1 h post treatment at 19:00 (n = 18, paired two-tailed t test, animals adapted to the appearance of test meal over the course of two weeks, males and females (male ♂ (black), female ♀ (grey)). (H) 1st hour (effect of treatment [F (1,98) = 14.70, p = 0.0002, std chow p > 0.9999, HFD p = 0.003, kcal p = 0.0067) and (I) 2nd hour (effect of treatment [F (1,102) = 1.65, p = 0.2014, std chow p = 0.9552, HFD p = 0.887, kcal p = 0.6080) food consumption of RXFP4^VMHDq^ mice in metabolic chambers, when mice had access to a choice between std chow and a HFD, post CNO (orange) or vehicle (black) treatment (n = 18 per group, mean ± SEM). Scale bars = 100 μm.

To assess whether the *Rxfp4*-expressing population in the VMH is involved in the feeding phenotype observed in RXFP4^wb−Dq^ mice, the effect of acute chemogenetic manipulation of RXFP4^VMH^ neuron activity on food intake was investigated. Male and female RXFP4^GCaMP3^ mice received bilateral VMH injections of Cre-dependent hM3Dq-expressing rAAVs (rAAV-hSyn-DIO-hM3D(G)q-mCherry) designed to preferentially target neurons, to produce RXFP4^VMHDq^ mice (Figure 7A). Targeting efficiency was subsequently determined by immunohistochemistry (Figure 7B), or in a subset of mice by fluorescent microscopy in live slices (see below). All mice demonstrated robust transduction that was limited to the target region. To confirm the functional activation of these RXFP4^VMHDq^ neurons, we generated *ex vivo* brain slices from a subset of RXFP4^VMHDq^ mice (Figure 7C). Calcium imaging demonstrated that *ex vivo* treatment with CNO activated mCherry/GCaMP3 positive RXFP4^VMHDq^, stimulating an increase in the frequency of calcium oscillations and an increase in GCaMP3 fluorescence in *Rxfp4*-expressing somas (Figure 7C and D).

The effect of chemogenetic activation of this cell population on food intake *in vivo* was studied in a crossover design. In chow fed male and female mice, and in line with RXFP4^wb−Dq^ animals, CNO treatment of RXFP4^VMHDq^ mice had no effect on standard chow intake at the onset of the dark phase (Figure 7E, as indicated). When animals were habituated to the appearance of a HFD or HPM, CNO resulted in a significant reduction in food intake at the onset of the dark phase (Figure 7F and G). When offered HFD and chow diet in parallel in metabolic cages, CNO significantly reduced intake of the HFD whilst intake of standard chow was not altered, resulting in an overall reduced caloric intake (Figure 7H) in male and female mice (Suppl. Fig. 5e and f). As seen with RXFP4^wb−Dq^ animals, this was a transient effect was no longer apparent in the 2nd hour post CNO administration (Figure 7I). CNO had no effect on energy expenditure or ambulatory activity in these RXFP4^VMHDq^ animals (Suppl. Fig. 5g and h).

## 3 Discussion

The *Rxfp4*-Cre mouse model generated in this study has enabled the identification of central *Rxpf4*-expressing cells, and aided the transcriptomic, functional and anatomical characterisation of a hypothalamic *Rxfp4*-expressing neuronal population. We show that *Rxfp4* is expressed in key feeding centres of the brain, including neurons of the VMH. *Ex vivo* CAMPER imaging revealed that INSL5 reduces cAMP levels in *Rxfp4*-expressing VMH neurons, and central administration of INSL5 into the VMH increased HFD and HPM intake in lean AL fed mice *in vivo*. Mimicking the native physiology of INSL5-RXFP4 via Di recapitulated these results, whereas chemogenetic cell activation via Dq suppressed HFD and HPM intake. Selective chemogenetic activation of *Rxfp4*-expressing cells in the VMH alone (targeted via Cre-dependent rAAV-Dq) also suppressed HFD and HPM intake, reflecting their position in brain circuits implicated in homeostatic and hedonic regulation of food intake [21]. Ablation of this population further demonstrated the importance of these neurones in governing long-term feeding behaviour. These data identify hypothalamic RXFP4 signalling as a key regulator of food intake and preference.

*Rxfp4* expression has been difficult to localise due to low mRNA expression levels and the lack of suitable verified antibodies. *Rxfp4* expression was previously reported in the colon [4] and in enteroendocrine tumor cell lines [22,23]. In contrast to our previous report [4], we detected *Rxfp4* mRNA in the hypothalamus (Figure 1G,H,J) and found substantial *Rxfp4*-dependent reporter expression in multiple brain regions, with distinct *Rxfp4*-expressing cell populations in the accessory olfactory bulb, RSC, VMHvl, and mammillary body (Suppl. Fig. 1). While it could be argued that this reflects lineage tracing from *Rxfp4*-positive precursor cells, the detection of *Rxfp4* mRNA by RT-qPCR and RNAscope, the activation of Cre-dependent rAAV-constructs when stereotactically injected into the adult VMH, the cAMP responsiveness of *Rxfp4*-positive cells to locally-perfused INSL5 in slice preparations, and the effect of stereotactically injected INSL5 on feeding behaviour, confirm active *Rxfp4*-promoter activity and consolidate active *Rxfp4* expression and function in the adult mouse brain (Figs. 1G, H and 7B).

To characterise the transcriptomic profile of hypothalamic *Rxfp4*-expressing cells we performed scRNAseq. Although some cells will have been lost and some genes may have exhibited altered expression during the cell dissociation and sorting process, the results allowed us to cluster *Rxfp4*-expressing cells into several subpopulations each characterised by a profile of cell-specific marker genes (Figure 2A and B). *Rxfp4* was identified in microglia and ependymocytes that potentially constitute the blood brain barrier, in addition to astrocytes, suggesting that INSL5 may additionally exert effects on non-neuronal cells [24]. Clustering of the *Rxfp4*-expressing neuronal population indicated a predominance of glutamatergic neurons with few GABAergic clusters (Figure 2D). Cluster 6 is of note given the expression of *Glp1r*, *Cckbr*, *Sstr1* and *Sstr2*, suggesting an overlap with other known appetite-modulating gut peptide receptors. Cocaine- and amphetamine-regulated transcript (*Cartpt*), expressed in cluster 3, and cannabinoid receptor 1 (*Cnr1*), expressed in clusters 1, 4 and 5, (Figure 2D, Suppl. Fig. 3) have also been implicated in energy homeostasis [25,26]. Cluster 1 displayed markers of a VMHvl *Esr1* population (*Esr1, Pgr, Tac1, Cckar, Rprm* and *Oxtr*) previously associated with food intake and energy expenditure [12,15,16]. However, chemogenetic activation of RXFP4^VMHDq^ neurons did not result in increased energy expenditure or ambulatory activity, contrasting with the previously described Esr1^VMHDq^ phenotype [16]. The role of RXFP4^VMH^ cells in feeding regulation is further suggested by the co-expression of the neuropeptide receptors *Mc4r* and *Nmur2.* MC4R activation has been linked to suppressed food intake through regulation of *Bdnf* expression in the VMH [27] – *Bdnf* was co-expressed in cluster 1 neurons in our dataset (Figure 2D). Acute administration of NMUR2 agonists have been shown to decrease feeding, with one agonist being somewhat selective to HFD intake regulation [28], mirroring RXFP4^VMHDq^ cell activation (Figure 7).

Mapping of the retrograde inputs to and anterograde projections from the *Rxfp4*-expressing cells in the VMH revealed a distinct neural circuitry surrounding this hypothalamic population. While we aimed to target the VMH specifically during stereotactic injections, disparities between current mouse brain atlases make it difficult to distinguish the VMH from the adjacent TN, which has also been implicated in feeding behaviour [13,29]. It is therefore possible that some *Rxfp4*-expressing cells in the TN were transfected by the viral vectors and acted as starter cells in these tracing experiments. Monosynaptic inputs to RXFP4^VMH^ cells were labelled predominantly from brain regions involved in homeostatic regulation of food intake such as the ARC, PVH and LHA (Figure 3F). Within the ARC, two populations of neurons have been intensely studied with regards to feeding regulation: pro-opiomelanocortin (POMC)/cocaine- and amphetamine-regulated transcript (CART)-expressing neurons inhibit food intake while Agouti-Related Peptide (AgRP)/neuropeptide Y (NPY)-expressing neurons stimulate food intake. These neurons integrate nutritional and hormonal signals from the periphery and send projections to multiple brain regions including the VMH [30]. Although the VMH is not thought to be the main target of arcuate POMC projections [31], the co-expression of *Mc4r* in the RXFP4^VMH^ neurons suggests that POMC neurons may be part of the ARC innervation of RXFP4^VMH^ neurons.

RXFP4^VMH^ cells predominantly project onto regions associated with reward and motivation-related behaviours (Figure 3C, Suppl. Fig. 3) such as the BNST, POA, CeA, paraventricular thalamic nucleus (PVT) and ventral tegmental area (VTA) [[32], [33], [34]], potentially underlying our finding that chemogenetic activation of *Rxfp4*-expressing cells in RXFP4^wb−Dq^ mice reduced an animal's drive to seek out and work for a highly palatable food reward (Figure 6G–I). Taken together, these data suggest that *Rxfp4*-expressing cells may influence motivation and reward-related behaviour via regulation of central reward signalling pathways. RXFP4^VMH^ cells also send projections to the PAG and PBN, two integration sites responsible for relaying sensory information between the forebrain and hindbrain and coordinating behaviour in response to various stimuli including metabolic, gustatory and nociceptive inputs [[35], [36], [37]]. This RXFP4^VMH^ projection map broadly recapitulates previously identified efferent projections from the VMH [[38], [39], [40]] and SST-expressing cells in the TN [13,38], as well as affarent projections to the VMH [41,42]. Interestingly, all retrograde-labelled input regions also received projections from RXFP4^VMH^ cells suggesting a high level of bidirectional connectivity within the RXFP4^VMH^ signalling network (Figure 3G). Similar bidirectional connectivity has been shown for an *Esr1*+ve VMHvl population [38]. The RXFP4^VMH^ neural network established in the present study suggests these cells may integrate metabolic and nutritional cues either directly or via other hypothalamic regions and regulate the reward system to influence ingestive behaviours. The low number of RXFP4^VMH^ input regions compared to projection regions suggests these cells may comprise an early node in this network.

Inhibition of *Rxfp4*-expressing cells via direct intra-VMH infusion of INSL5 or whole body Di acutely increased intake of both a HFD and a HPM in the home cage and when animals were offered a choice of standard laboratory chow and HFD in metabolic cages, without altering energy expenditure and activity in both male and female mice (Figs. 1N, O, 5, Suppl. Fig. 5a and b). This is consistent with our previous demonstration that INSL5 administration increased food intake [4,5]. Activation of Di receptors should, at least in part, mimic the Gα_i/o_-coupling of RXFP4 [2]. By contrast, activation of *Rxfp4*-expressing cells with Dq produced a robust suppression of HFD and HPM intake (Figure 6). Targeting the RXFP4^VMH^ population with an rAAV-Dq reporter recapitulated the findings with the whole body-Dq approach (Figure 7). Whilst any of the identified *Rxfp4*-expression sites could participate in the feeding phenotype of the global DREADD reporter mice, the VMH-specific rAAV-Dq reporter phenotype indicates that this hypothalamic population either underlies or at least contributes to the observed anorexigenic effects in the RXFP4^wb−Dq^ model.

Our data suggest that activation of *Rxfp4*-expressing cells in the VMH suppresses the consumption and drive to work for calorie dense HFD and HPM. The importance of the VMH in the regulation of feeding and metabolism has been disputed (reviewed in [43]). Initial studies suggested the VMH might be a “satiety centre”, as VMH-lesioned rats, particularly females, over-consumed a HFD when fed AL [44], despite being seemingly less willing to work for food on a fixed ratio lever-pressing paradigm [45]. The observed hyperphagia has subsequently been linked to additional damage to adjacent hypothalamic structures (see King 2006 [43] for discussion), while the reduced motivation was not observed when rats were trained pre-operatively, suggesting that the VMH lesion may have altered “trainability” rather than feeding motivation [43,46]. Lesioning studies, whilst informative, lack cellular precision and damage neural connections to other feeding centres within the brain. More recent work employing immunohistochemistry, RNA sequencing, chemogenetics and neuronal projection mapping, has demonstrated that the VMH consists of anatomical subdivisions made up of distinct cell populations [15,47,48]. Functional studies suggest neurons in the central and dorsomedial VMH regulate feeding, energy expenditure and glucose homeostasis [29,49], while the VMHvl is more frequently implicated in the control of social and sexual behaviours [48,50,51]. However, several studies have demonstrated the involvement of VMHvl neurons in energy expenditure and feeding behaviour [12,52]. Increases in physical activity have been observed following chemogenetic activation of NK2 homeobox transcription factor 1 (*Nkx2-1*)-expressing neurons in the VMHvl of female rats, while knockout of *Nkx2-1* in the VMHvl leads to decreased physical activity and thermogenesis [53]. Furthermore, chemogenetic activation of *Esr1*-expressing VMHvl neurons was found to stimulate physical activity and thermogenesis in both sexes [16]. *Esr1* signalling in the VMHvl was previously demonstrated to influence food intake, energy expenditure and glucose tolerance and VMHvl-restricted knockdown of *Esr1* resulted in increased food intake, decreased physical activity and thermogenesis, reduced glucose tolerance and obesity in female rats [12]. Activation of RXFP4^VMHDq^ neurons reduced HFD and HPM intake but had no effect on chow intake, energy expenditure or ambulatory activity in both male and female mice (Figure 7, Suppl. Fig. 5e–h). This, alongside the transcriptomic profile and neural circuitry of RXFP4^VMH^ neurons, suggests that these neurons comprise a distinct VMH population modulating food intake and preference based on the rewarding aspects of food rather than the homeostatic food intake or energy expenditure responses observed during chemogenetic manipulation of other VMH populations.

We recognise several limitations to this study. First, the exact classification of *Rxfp4*-expressing brain regions is difficult given the disparities between current brain atlases. Whilst we aimed to target *Rxfp4*-expressing cells in the VMH, the inexact nature of stereotactic injections may have resulted in additional targeting of neurons in adjacent LHA and TN regions, which may have contributed to the food intake phenotype of RXFP4^VMHDq^ mice. The fact that only a subset of RXFP4^CAMPER^ neurons responded to INSL5 in acute slices likely reflects technical limitations of this preparation and the difficulty in recording reductions in cAMP downstream of Gi. The similarities in feeding outcomes of intraparenchymal INSL5 in wildtype mice and CNO application in RXFP4^VMHDq^ mice together with RNAscope confirmation of *Rxfp4*-Cre labelled VMH neurons makes the alternative explanation of substantial additional off target Cre-expression unlikely, but we cannot fully rule this out. It is also uncertain whether INSL5 is the endogenous ligand acting on *Rxfp4*-expressing cells in the brain. We have previously been unable to identify *Insl5*-expressing cells in the mouse brain [5] and there is no evidence that INSL5 can cross the blood brain barrier. In addition, relaxin-3 also activates RXFP4 [2] and is expressed in the mouse brain [54], hence it is possible that relaxin-3 is involved in central RXFP4 action. Intracerebroventricular injection of relaxin-3 stimulates food intake in rats [[55], [56], [57]], but given that *Rxfp4* is a pseudogene in rats, this orexigenic action is likely due to activation of RXFP3 [54,58]. Relaxin-3 has not been demonstrated to be orexigenic in mice, but both relaxin-3 and *Rxfp3* knockout mice demonstrate dark phase hypoactivity (assessed via voluntary home-cage wheel running) [59,60], potentially reflecting reduced motivational drive. Whilst an RXFP3-selective antagonist reduced motivated food seeking behaviour and palatable food intake in wild-type but not *Rxfp3* knockout mice, icv injection of a synthetic RXFP3-agonist, which cross reacts with RXFP4, failed to stimulate chow or palatable food intake in wild type mice [[61], [62], [63]]. However, our own previous attempts to increase food intake by injecting INSL5 icv also failed to produce significant results [4] contrasting with the results after intraparenchymal injection presented here. The source of a central RXFP4 ligand in the mouse CNS remains obscure and should be addressed in future research. The viral tracing techniques used to decipher the RXFP4^VMH^ neuronal network also have some limitations. In the retrograde tracing experiments, it was difficult to detect rAAV-GFP immunoreactive cells, making it hard to confirm the exact starter cells in this experiment. Furthermore, while the ChR2-mCherry construct is preferentially targeted to axon terminals, it is possible that some of the mCherry-labelled fibres in adjacent regions, such as the ARC, are in fact dendrites [64] which may underlie our inability to detect retrograde-only labelled regions. Nevertheless, we have been able to identify distinct regions that project onto and receive projections from *Rxfp4*-expressing cells in the VMH that connect these cells to known feeding-related neural networks. The data generated following ablation of RXFP4^VMH^ neurons are somewhat unexpected given native INSL5-RXFP4 physiology, the INSL5 effects on cAMP in RXFP4^CAMPER^ cells in *ex vivo* brain slice imaging and the DREADD-based feeding phenotypes, which are all consistent with the model that RXFP4-dependent inhibition of RXFP4^VMH^ neurons results in increased HFD and HPF intake. The reduced HFD and HPM intake after ablation of RXFP4^VMH^ neurons and the reduced weight gain of HFD fed RXFP4^VMHKO^ animals likely reflects loss of the plethora of neurotransmitters and receptors of these neurons, exemplified by the scRNA-seq data, and the chronic disruption to the neural circuits surrounding these neurons, possibly also affecting other behaviours, given that the VMHvl also modulates social behaviour and aggression [15] even though we did not observe any obvious behavioural abnormalities. Finally, most of the functional data included in this manuscript reflect ablation, activation and inhibition of *Rxfp4*-expressing neurons rather than of the receptor itself. Nevertheless, the phenotypes observed highlight that these neurons are involved in regulating food intake and that manipulation of these neurons, via RXFP4, could affect long-term feeding behaviour.

In summary, we have characterised a previously unrecognised population of ventromedial hypothalamic cells that express *Rxfp4* in mice, identified projections in homeostatic and hedonic feeding centres in the CNS, and demonstrated that acute manipulation of these cells modulates HFD/HPM intake without affecting chow intake or energy expenditure. Together, these findings suggest *Rxfp4*-expressing neurons in the VMH are key regulators of food preference and represent a target for the modulation of feeding behaviour.

## 4 Methods

### 4.1 Animals

All experiments were performed under the UK Home Office project licences 70/7824 and PE50F6065 in accordance with the UK Animals (Scientific Procedures) Act, 1986 and approved by the University of Cambridge Animal Welfare and Ethical Review Body. All mice were from a C57BL/6 background and were group-housed and maintained in individual ventilated cages with standard bedding and enrichment in a temperature and humidity controlled room on a 12 h light:dark cycle (lights on 7:00) with ad libitum (AL) access to food (Scientific Animal Food Engineering) and water unless otherwise stated. Groups were randomised by body weight and the researcher was blinded to treatment.

### 4.2 Mouse models

To express Cre recombinase under the control of the *Rxfp4* promoter, we replaced the sequence between the start codon and the stop codon in the single coding exon of *Rxfp4* in the murine-based BAC RP24-72I4 (Children's Hospital Oakland Research Institute) with iCre [65] sequence using Red/ET recombination technology (GeneBridges) (Figure 1A). The resulting BAC was used to create BAC-transgenic mice – of four initial founders, two passed the transgene to their offspring; both resulting lines showed similar Cre-reporter expression and one line was used throughout this manuscript. Several Cre-reporter transgenes, in which expression is only activated after removal of a fxSTOPfx cassette, were used, resulting in expression of EYFP [66], hM3Dq, hM4Di [67], or GCaMP3 [68], respectively.

### 4.3 Viral injections

Viral injections were performed in male and female *Rxfp4*-Cre mice aged between 9 and 16 weeks. The surgical procedure was performed under isoflurane anaesthesia, with all animals receiving Metacam prior to surgery. Mice were stereotactically implanted with a guide cannula (Plastics One) positioned 1 mm above the VMH (A/P: −1.7 mm, D/V: −4.5 mm, M/L: +/− 0.75 mm from bregma). Bevelled stainless steel injectors (33 gauge, Plastics One) extending 1 mm from the tip of the guide were used for injections. For anterograde viral tracing experiments, 200 nL Cre-dependent rAAV-DIO-ChR2-mCherry (Addgene, 20,297-rAAV8, 1.9 × 10^1^³ vg/ml) was injected unilaterally at 75 nL/min. Mice were culled three weeks after injection. For retrograde viral tracing experiments, AVV2-FLEX-TVAeGFP-2A-oG (rAAV2-TVAeGFP-oG) and Rabies-ΔG-EnvA-mCherry (Rab-ΔG-EnvA-mCherry) viruses were generated by Ernesto Ciabatti (MRC Laboratory of Molecular Biology, Cambridge). Mice were injected unilaterally with 200 nL Cre-dependent AVV2-TVAeGFP-oG (1 × 10^12^ vg/mL) at 75 nL/min followed by 500 nL Rab-ΔG-EnvA-mCherry (2 × 10^9^ iu/mL) at 75 nL/min three weeks later. Mice were culled seven days after the second injection. For the RXFP4^VMHKO^ studies, 200 nL Cre-dependent ssAAV9-hEF1α-dlox-DTA (rev)-dlox-WPRE-hGHp(A) (rAAV-DTA: ETH VVF, 6.2 × 10^12^ vg/mL) or Cre-dependent AAV2-hSyn-DIO-mCherry (rAAV-mCherry: Addgene, 50,459-AAV2, 4 × 10^12^ vg/mL) was injected bilaterally at 75 nL/min and mice were allowed 2 weeks recovery prior to testing. For phenotyping experiments, 200 nL Cre-dependent rAAV8-hM3D(G)q-mCherry (Addgene 44,361-rAAV8, 4 × 10^12^ vg/mL) was injected bilaterally at 50 nL/min and mice were allowed 2 weeks recovery prior to testing.

### 4.4 Intra-VMH infusion

Mice were stereotactically implanted with a guide cannula (Plastics One) positioned 1 mm above the VMH (A/P: −1.7 mm, D/V: −4.5 mm, M/L: ±0.75 mm from bregma). Bevelled stainless steel injectors (33 gauge, Plastics One) extending 1 mm from the tip of the guide were used for infusions in free-moving mice. Animals was given 1 week to recover. Mice were given AL access to standard (std) chow.

### 4.5 Food intake

Food intake studies were performed in a cross-over design, on age-matched groups, a minimum of 72 h apart. Mice were administered vehicle or INSL5 (5 μg/animal, Phoenix Biotech, 035-40 A) at 12:00-15:00 following a 2 h fast. Food was weighed 1 h post-injection. For experiments assessing the effect of global RXFP4 Di and Dq activation, animals were singly housed prior to the experiment. Mice were administered 1 mg/kg CNO (Sigma, C0832) ip or an equivalent volume of vehicle containing a matched concentration of DMSO (Sigma, D2650). For light phase activation, animals were injected with vehicle or CNO at 11:00 (±30 min) following a 2 h fast. Food was weighed at 1 h post-injection. For dark phase activation, animals were injected with vehicle or CNO at 19:00 at the onset of the dark phase following a 2 h fast. Food was weighed at 1 h post-injection. In trials with a high fat diet (HFD, Open Source Diets, D12451) and a highly palatable meal (HPM, liquid Ensure, Abbott Laboratories, 353-3601), mice were habituated to the appearance of the test meal for 1 h (5 days per week) for two weeks prior to testing.

### 4.6 Operant chambers

Twelve male RXFP4^wb−Dq^ mice (weighed 3 times weekly) were food restricted to maintain 95% body weight for two weeks prior to training and testing in standard mouse Bussey-Saksida touchscreen chambers (Campden Instruments Ltd, Loughborough, UK). Training and testing procedures were conducted as previously described [69]. Briefly, mice were trained to touch a screen for a reward (HPM, 20 μL) under a fixed ratio (FR) schedule for 2 weeks, progressing from FR1 to FR5 (training deemed successful when the animal earnt 30 rewards within 1 h), followed by testing. Mice then progressed to progressive ratio (PR, increment +4 i.e. 1, 5, 9, 13, etc.), where the breakpoint was defined as the last reward earned before 5 min elapsed without operant response. Following testing of the breakpoint, mice progressed to the effort related choice schedule (ERC) - mice were trained on FR8, with the addition of standard chow to the operant arena. Once animals successfully earned 30 rewards within 60 min, testing was undertaken. The 60-minute training and testing sessions took place at the same time each day (between 10:00 and 13:00).

## 5 Metabolic cages

Animals were acclimated to metabolic cages prior to study and data collection. Oxygen consumption and carbon dioxide production were determined using an indirect calorimetry system (Promethion, Sable Systems, Las Vegas, NV). The system consisted of 8 metabolic cages (similar to home cages), equipped with water bottles and food hoppers connected to load cells for continuous monitoring, in a temperature and humidity-controlled cabinet. The respiratory exchange ratio (RER) and energy expenditure (via the Weir equation) were calculated, whilst ambulatory activity was determined simultaneously. Raw data were processed using ExpeData (Sable Systems). Animals were exposed to standard chow and a HFD during metabolic assessment as indicated.

### 5.1 CAMPER and calcium imaging

Mice were anesthetized using sodium pentobarbital at a dose of 180 mg/kg (Dolethal, Vetoquinone). A laparotomy was then performed, the heart exposed, and the animals were transcardially perfused with oxygenated High-Mg^2+^/low Ca^2+^ ice-cold artificial cerebrospinal fluid (perfusion solution; composition in mM: 2.5 KCl, 200 sucrose, 28 NaHCO_3_, 1.25 NaH_2_PO4, 8 Glucose, 7MgCl_2_, 0.5 CaCl_2_; pH 7.4). The brain was extracted from the skull and immersed in oxygenated perfusion solution. 250 μm thick slices of the hypothalamus containing the VMH were cut on a Vibratome (Leica) and placed in a recovery solution (in mM: 3 KCl, 118 NaCl, 25 NaHCO_3_, 1.2 NaH2_P_O_4_, 2.5 Glucose, 7 MgCl_2_, 0.5 CaCl_2_; pH 7.4) at 34 °C for 30 min. Sections were then transferred to standard ACSF (in mM: 3 KCl, 118 NaCl, 25 NaHCO_3_, 10 Glucose, 1MgCl_2_, CaCl_2_; pH7.4) at room temperature for 1 h before recording. All solutions were continuously bubbled with 95% O_2_/5% CO_2_.

Imaging was performed using a Zeiss Axioskop2 FS Plus microscope. Slices were immobilised in a glass bottom chamber using a slice anchor (Warner instruments) and imaged using a 40× water immersion objective. For CAMPER imaging, RXFP4^CAMPER^ neurons of the VMH were excited using a LED light source (Dual Optoled, Cairn Research) at 435 for 50 ms every 3 s and CFP and YFP emission were simultaneously recorded after passing through an optosplit (Cairn; 465–505 and 525–600 nm, respectively). For Ca^2+^ imaging, RXFP4^GCaMP3^ neurons were excited at 470 nm for 75 ms every 2 s and emission from 500 to 550 nm was captured. Images were captured using a charged-coupled camera (Evolve 512 Delta, Photometrics), acquisitions were saved and data logged using Metamorph software (Molecular Devices). Slices were continuously perfused with room temperature standard ACSF at a flow rate of 1 mL per minute. All drugs were added directly in standard ACSF. CNO (15 μM, C0832), IBMX (100 μM, I7018) and forskolin (10 μM, F6886) were purchased from Sigma. INSL5 (100 nM, 035-40 A) was purchased from Phoenix Biotech.

Regions of interest (ROIs) and an area determined for background fluorescence were outlined for each image and the mean pixel intensity extracted for each ROI. The mean intensity of the background was subtracted from each ROI. For CAMPER imaging, the fluorescence energy transfer ratio (ΔFRET) of the mTurquoise FRET donor (CFP) and Venus FRET acceptor (YFP) was calculated and is presented on a reverse axis. INSL5 responses were measured by calculating the percentage change in ΔFRET between the maximum IMBX response and the maximum IBMX + INSL5 response, both compared to the maximum IBMX + Forskolin response. Changes of more than 20% of the IBMX + Forskolin responses were considered responses. For Ca^2+^ imaging, fluorescence intensity of the GCaMP3 is presented as ΔF/F_0_ with F_0_ being the mean intensity for 5 min before stimulus and ΔF the fluorescence intensity F minus F_0_. Responses were observed on raw and ΔF/F_0_ traces and quantified by calculating areas under the curve (AUCs) over 5 min before the stimulus for the ACSF condition and over 5 min after the stimulus for the treated conditions. Cells were considered activated by stimuli if the AUC increased by 25% after treatment compared to the AUC before treatment.

### 5.2 Immunohistochemistry

Colonic tissues were fixed in 4% paraformaldehyde (PFA; Alfa Aeser, J61899), dehydrated in 15% and 30% sucrose, and frozen in OCT embedding media (CellPath, Newtown, U.K.). Cryostat-cut sections (8–10 μm) were mounted directly onto poly-l-lysine-covered glass slides (VWR, Leuven, Belgium) by the Institute of Metabolic Science Histology Core. Slides were incubated for 1 h in blocking solution containing 10% goat or donkey serum. Slides were stained overnight at 4 °C with primary antisera (table 1) in PBS/0.05% Triton X-100/10% serum. Slides were washed with blocking solution and incubated with appropriate secondary antisera (donkey or goat Alexa Fluor® 488, 546, 555, 633 or 647; Invitrogen) diluted 1:400 and Hoechst diluted 1:1500 for 1 h. Control sections were stained with secondary antisera alone. Sections were mounted with Prolong Gold (Life Technologies) or hydromount (National Diagnostics, Atlanta, Georgia, USA) prior to confocal microscopy (Leica TCS SP8 X, Wetzlar, Germany). Quantification of cell number was performed using Leica Application Suite X and Image J.

**Table 1:** Primary antisera used for immunohistochemistry.

Antigen	Raised In	Concentration	Source
GFP	Goat	1:1000 (unless specified)	Abcam, 5450
DSRed (RFP and mCherry)	Rabbit	1:1000	Takara Bio Clontech, 632,496
NeuN	Mouse	1:1000	Millipore, MAB377

Brain tissue was collected from perfusion fixed mice as previously described [70]. Animals were anaesthetised with dolethal sodium pentobarbital solution at 125 mg/kg ip (in saline) and transcardially perfused with heparinised 0.1 M phosphate buffered saline (1xPBS) followed by 4% PFA in PBS. Brains were extracted and post-fixed in 4% PFA for 24 h at 4 °C then transferred to 30% sucrose solution at 4 °C for 48 h. Brains were sectioned coronally from olfactory bulb to the spinomedullary junction at 25 μm using a freezing microtome and stored in cryoprotectant medium. For diaminobenzidine (DAB) staining, antigen retrieval was used for all experiments prior to antibody incubation. Sections were incubated in 10 mM sodium citrate (Alfa Aesar, A12274) at 80 °C for 10 min then washed in PBS. Sections were incubated in 0.5% hydrogen peroxide (Sigma, H1009) in milliQ water for 15 min then washed in PBS. Sections were blocked with 5% donkey serum in 0.3% Tween20 (VWR, 437082Q) in PBS (PBST) for 1 h at room temperature, then incubated with GFP antiserum (1:4000; ab5450, Abcam) in blocking solution overnight at 4 °C. After washing in 0.1% PBST, sections were incubated with biotinylated anti-goat IgG (1:400; AP180B, Millipore) in 0.3% PBST for 1.5 h at room temperature, followed by a 1 h incubation with streptavidin conjugated to horseradish peroxidase (Vectastain Elite ABC kit, Vector Laboratories, PK-6100) and developed by DAB substrate (Abcam, ab94665). Sections were washed in PBS, prior to dehydration in ethanol and xylene, then mounting/coverslipping with Pertex mounting medium (Pioneer Research Chemicals Ltd., PRC/R/750). For immunofluorescent staining, slices were washed in PBS, prior to blocking for 1 h in 5% donkey serum then incubation with primary antisera (Table 1) in blocking solution overnight at 4 °C. Slices were washed in and incubated with the appropriate secondary antisera (Alexa Fluor® 488 or 555; Invitrogen) diluted 1:500 for 2 h at room temperature. Following washing, mounted sections were coverslipped on superfrost slides using Vectashield (Vector Laboratories, H-1400-10). Slides were imaged using an Axio Scan. Z1 slide scanner (Zeiss) and confocal microscope (Leica TCS SP8 X, Wetzlar, Germany) with a 20× or 40× objective as indicated. Images were analysed in Halo Image Analysis Software (Indica Labs) and ImageJ.

### 5.3 Tissue extraction and reverse transcription-quantitative polymerase chain reaction (RT-qPCR)

Animals were culled by cervical dislocation. Brain and colon tissues were extracted and frozen immediately on dry ice. For pancreatic islet extraction, pancreases were injected with collagenase V (0.5 mg/mL) and digested at 37 °C. Islets were hand-picked into HBSS containing 0.1% wt/vol. fatty acid-free BSA. Each pancreas yielded approximately 150–300 islets which were pooled. RNA from whole tissue segments was extracted using TRIzol Reagent (Sigma, T9424) according to manufacturer's instructions. RT-qPCR was performed and analysed as described in [71] using probes Rxfp4: Mm00731536_s1 and b-actin: Mm02619580_g1 (Applied Biosystems). Mean, SEM and statistics were obtained for the ΔCT data and converted to relative expression levels (2ΔCT) for presentation only.

### 5.4 Dissociation, fluorescence-activated cell sorting (FACS) and single cell RNA sequencing

For hypothalamic samples, single cell suspensions were prepared and pooled from twelve female *Rxfp4*-Cre x EYFP mice (biological replicates) as previously described [70]. Briefly, mice were sacrificed by cervical dislocation and the hypothalamus dissected into Hibernate-A medium (ThermoFisher, A1247501) supplemented with 0.25% GlutaMAX (ThermoFisher, 35050061) and 2% B27 (ThermoFisher, A1895601). Tissue was digested in Hibernate-A medium without calcium (BrainBits) containing 20 U/mL Papain (Worthington) and 1% GlutaMAX for 30 min at 37 °C under agitation (Thermomixer, 500 rpm). After digestion, tissue was triturated in Hibernate-A medium with 3.5 U/mL DNase I (Sigma, D4263). The trituration supernatant was loaded on top of a BSA gradient prepared in Hibernate-A medium, spun for 5 min at 300 rcf, and the pellet was resuspended in Hibernate-A medium +1% BSA. The cell suspension was filtered through a 70 μm cell strainer into a fresh tube. Fluorescence-activated cell sorting was performed using an Influx Cell Sorter (BD Biosciences, San Jose, CA, USA). Cell gating was set according to cell size (FSC), cell granularity (SSC), FSC pulse-width for singlets, fluorescence at 488 nm/532 nm for EYFP and 647/670 nm for nuclear stain with DraQ5 (Biostatus, Shepshed, Leicester, UK) to exclude cellular debris, aggregates and dead cells. Cells were sorted directly into individual wells of 96-well plates containing lysis buffer. 384 YFP-positive cells were isolated and processed using a Smart-Seq2 protocol [72]. Libraries were prepared from ∼150 pg of DNA using the Nextera XT DNA preparation kit (Illumina, FC-131-1096) and Nextera XT 96-Index kit (Illumina, FC-131-1002). Pooled libraries were run on the Illumina HiSeq 4000 at the Cancer Research UK Cambridge Institute Genomics Core. Sequencing reads were trimmed of adapters, aligned to the *Mus musculus* genome (GRCm38, Ensembl annotation version 101) using STAR (v2.7.3a), and raw counts generated using FeatureCounts (Subread v2.0.0). Downstream analyses were performed using the Seurat R package (v4.0.1). Samples were included in the final analyses only if they met all of the following criteria: (a) unique reads >50%, (b) reads assigned to exons >20%; (c) number of genes with (FPKM>1) > 3000; (d) total number of unique genes >200. Genes were included in the final analyses if they were detected in at least 3 samples. Marker genes were identified for clusters using the FindAllMarkers function in the Seurat package; the top 20 gene markers were cross referenced against other bulk and single cell RNAseq databases to assign cell type. Neuronal clusters were identified in a similar fashion using appropriate marker genes.

### 5.5 RNAscope

Detection of Mouse *Rxfp4* was performed on fixed, frozen sections using Advanced Cell Diagnostics (ACD) RNAscope® 2.5 LS Reagent Kit-RED (Cat No. 322150), RNAscope® LS 2.5 Probe-Mm-Rxfp4 (Cat No. 317588) (ACD, Hayward, CA, USA). To prepare the sections, animals were anaesthetized with sodium pentobarbital solution (50 mg/kg in saline) and transcardially perfused with PBS followed by 4% PFA in PBS. Brains were extracted and post-fixed in 4% PFA for 24 h before being transferred to 25% sucrose for 24 h at 4 °C. Brains were embedded in OCT compound, frozen in a Novec 7000 (Sigma)/dry ice slurry and stored at −80 °C. 16 μm cryosections containing the hypothalamus were prepared on a Leica CM1950 cryostat (Wetzlar, Germany) at −12 °C and stored at −20 °C until required.

Slides were thawed at room temperature for 10 min before baking at 60 °C for 45 min. The sections were then post-fixed in pre-chilled 4% PFA for 15 min at 4 °C, washed in 3 changes of PBS for 5 min each before dehydration through 50%, 70%, 100% and 100% ethanol for 5 min each. The slides were air-dried for 5 min before loading onto a Bond Rx instrument (Leica Biosystems). Slides were prepared using the frozen slide delay prior to pre-treatments using Epitope Retrieval Solution 2 (Cat No. AR9640, Leica Biosystems) at 95 °C for 5 min, and ACD Enzyme from the LS Reagent kit at 40 °C for 10 min. Probe hybridisation and signal amplification were performed according to manufacturer's instructions, with the exception of increased Amp5 incubation time at 30 min. Fast red detection of mouse *Rxfp4* was performed on the Bond Rx using the Bond Polymer Refine Red Detection Kit (Leica Biosystems, Cat No. DS9390) with an incubation time of 20 min. Slides were then removed from the Bond Rx and were heated at 60 °C for 1 h, dipped in xylene and mounted using EcoMount Mounting Medium (Biocare Medical, CA, USA. Cat No. EM897L). Sections were imaged on a Slide Scanner Axio Scan. Z1 microscope (Zeiss) using a 40× air objective. Three z-stack slices spanning 1.5 μm were combined into an extended depth of field image (ZEN 2.6, Zeiss). The CZI files were read into Halo Image Analysis Software (Indica Labs).

### 5.6 Statistical analysis

Data were plotted using GraphPad Prism 7/8/9 software (GraphPad Software, Inc). Statistical analysis was performed by paired Student's t-tests, one-way ANOVA with multiple comparisons or two-way ANOVA with multiple comparisons, and ANCOVA, as indicated. N represents biological replicates. Sample size was computed based on pilot data and previously published data. Data are presented as mean ± SEM and probabilities of p < 0.05 were considered statistically significant in all tests.

## Author contributions

JEL, ORMW, FMG and FR designed the research studies. JEL, ORMW, DN, CB, LB and AEA conducted experiments. SJK and BG conducted the SmartSeq2 protocol and library preparation for single-cell RNA-sequencing and CAS led the bioinformatic analysis. EC and MT provided the rabies and rAAV viruses for retrograde viral tracing. JAT prepared the tissues and gave guidance for RNAscope. DH and DB co-supervised ORMW and BUP helped with the initial behavioural cage experiments. FR developed the transgenic models. JEL, ORMW, DN, FMG and FR wrote the manuscript. All authors revised and approved the final draft.

## Data Availability

RNASeq data available via GEO upon publication
