# Supplementary material for: Relaxin/insulin-like family peptide receptor 4 (Rxfp4) expressing hypothalamic neurons modulate food intake and preference in mice
Source: Mol Metab. 2022 Sep 30;66:101604. doi: 10.1016/j.molmet.2022.101604 (PMC9579047; doi:10.1016/j.molmet.2022.101604)
Supplement: Multimedia component 1 [file mmc1.pptx]

## Slide 1
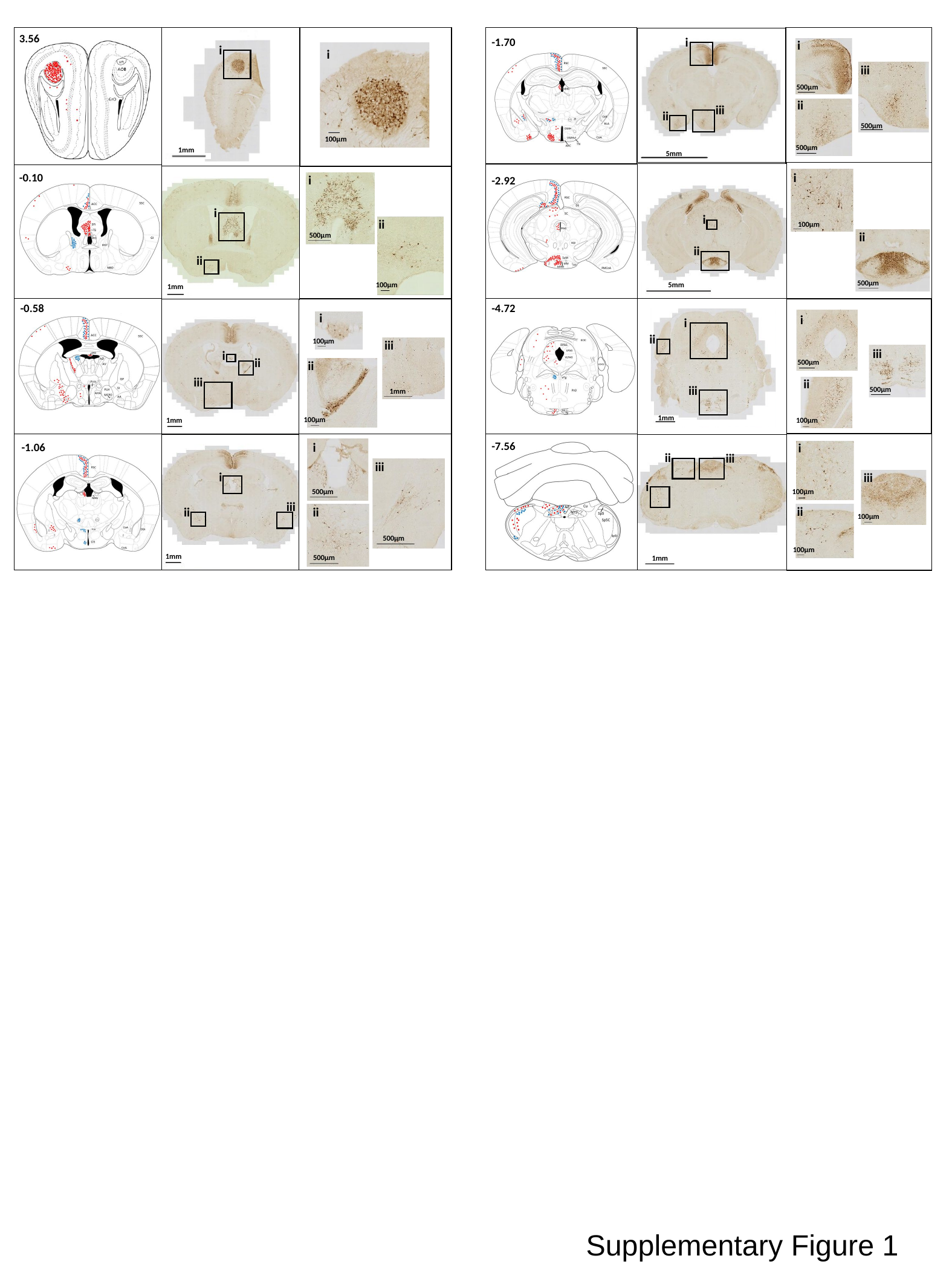

3.56
i
-1.70
i
i
i
iii
500µm
ii
iii
ii
500µm
100µm
500µm
1mm
5mm
i
-0.10
i
-2.92
i
i
ii
100µm
ii
500µm
ii
ii
500µm
100µm
5mm
1mm
-4.72
-0.58
i
i
i
ii
100µm
iii
iii
i
ii
500µm
ii
iii
ii
iii
500µm
1mm
1mm
100µm
1mm
100µm
i
-7.56
-1.06
i
ii
iii
iii
i
iii
i
500µm
100µm
iii
ii
ii
ii
100µm
500µm
100µm
1mm
500µm
1mm
Supplementary Figure 1

## Slide 2
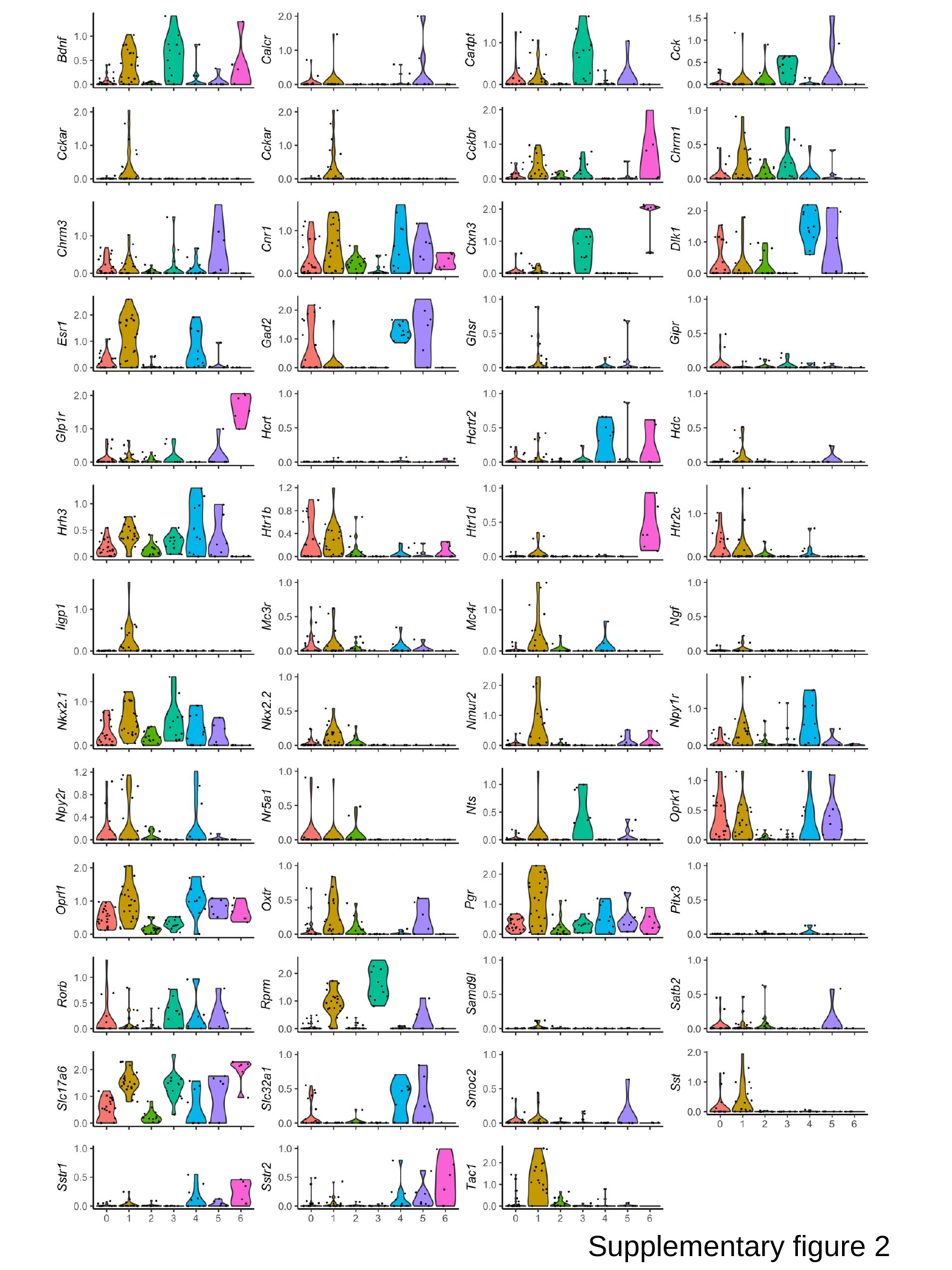

B
Supplementary figure 2

## Slide 3
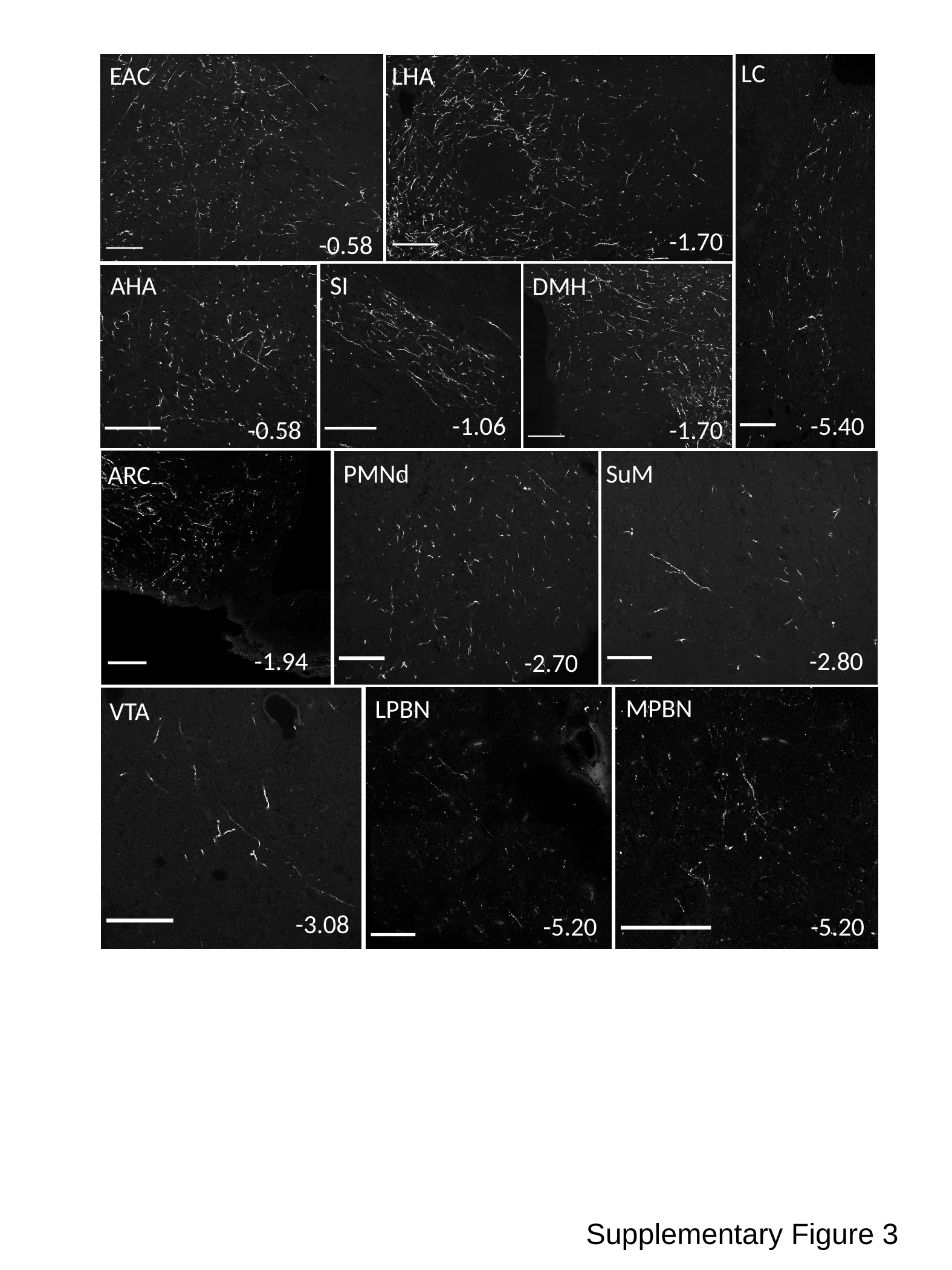

LC
EAC
LHA
LHA
-1.70
-0.58
SI
AHA
DMH
-1.06
-5.40
-1.70
-0.58
PMNd
SuM
ARC
-2.80
-1.94
-2.70
MPBN
LPBN
VTA
-3.08
-5.20
-5.20
Supplementary Figure 3

## Slide 4
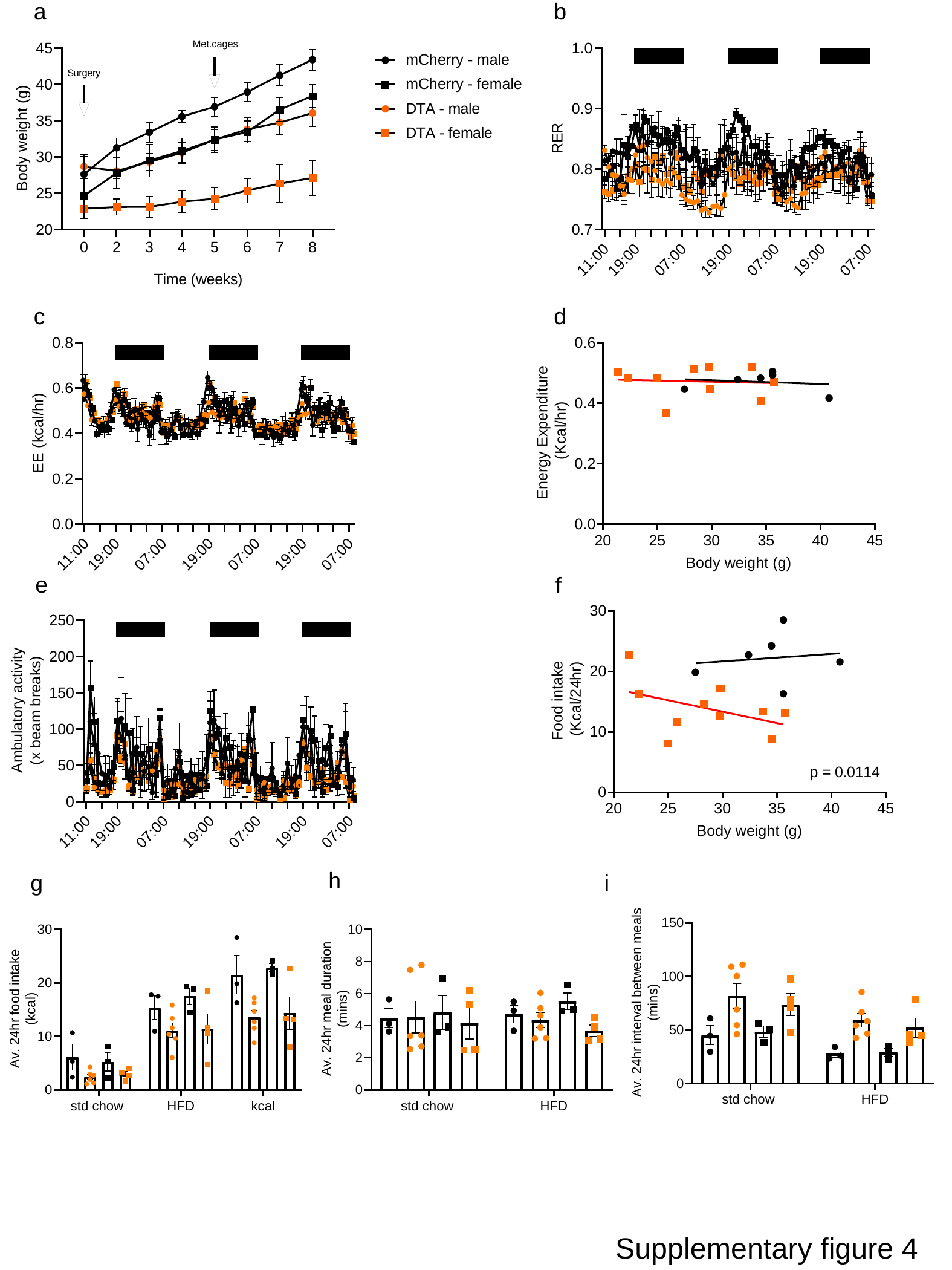

a
b
c
d
e
f
h
g
i
Supplementary figure 4

## Slide 5
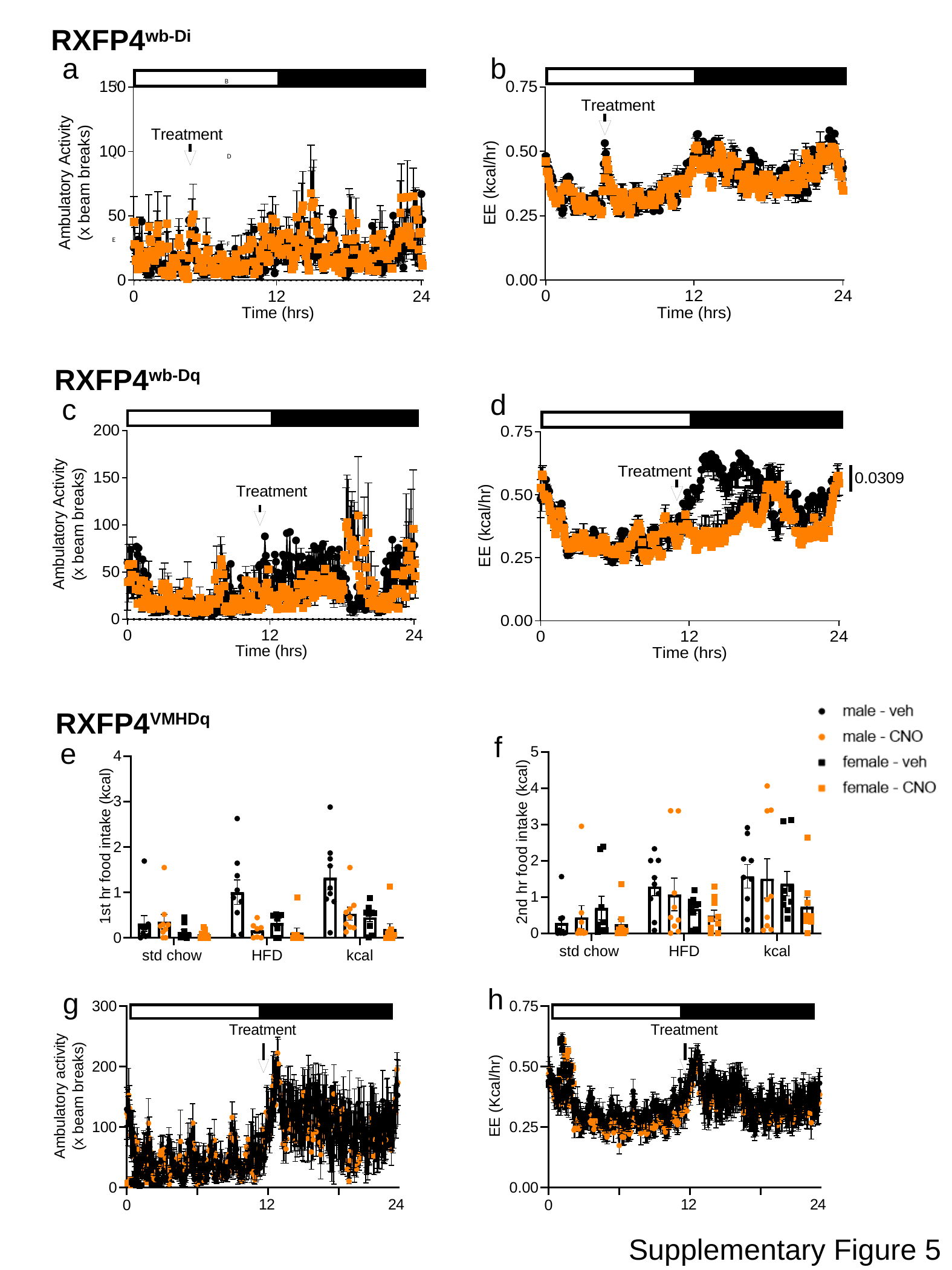

RXFP4wb-Di
b
a
B
A
C
D
E
F
RXFP4wb-Dq
d
c
RXFP4VMHDq
f
e
h
g
Supplementary Figure 5
